# Review of *Anasillomos* Londt, 1983 with the description of a new species (Insecta: Diptera: Asilidae)

**DOI:** 10.3897/BDJ.3.e4652

**Published:** 2015-03-05

**Authors:** Torsten Dikow

**Affiliations:** ‡ National Museum of Natural History, Smithsonian Institution, Washington, DC, United States of America

**Keywords:** Afrotropical, assassin flies, identification keys, cybertaxonomy, data sharing

## Abstract

The southern African assassin-fly genus *Anasillomos* Londt, 1983 is reviewed. A new species, *Anasillomos juergeni* sp. n., is described from the Namib desert and represents the second species in the genus. Descriptions/re-descriptions, photographs, and identification keys are provided to aid in the identification. Distribution, occurrence in biodiversity hotspots *sensu* Conservation International, and seasonal incidence are discussed.

## Introduction

**Figure 1.**
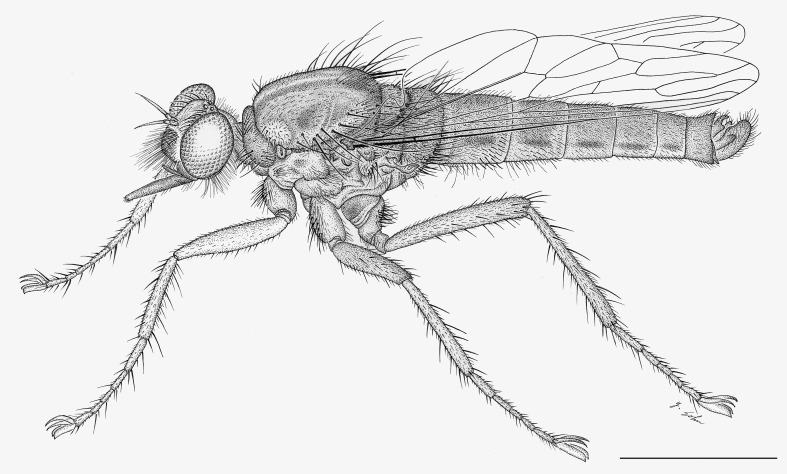
*Anasillomos juergeni* sp. n. holotype male (USNMENT00832201). Scale line = 5 mm.

The genus *Anasillomos* Londt, 1983 (Fig. 1​) was originally described from eastern Botswana and Namibia based on only seven specimens representing a single species (Londt 1983). *Anasillomos chrysopos* Londt, 1983 therefore exhibited a large geographic range with little morphological variation except that Londt (1983) mentioned that a single female specimen from Gobabeb in the central Namib desert showed variation in the bristliness and coloration. Later, Dikow and Londt (2000) recorded *Anasillomos chrysopos* for the first time in South Africa — from Carnarvon (Northern Cape Province) in the central Karoo — based on a single female specimen. The authors also mentioned four recently collected female specimens from southern Namibia, which were believed to be distinct morphologically, but not described as a new species due to the lack of males.

This review was instigated by the collection of 10 specimens of *Anasillomos* at the Gobabeb Research and Training Centre (www.gobabebtrc.org) by the author in February 2012. Even though only a single male specimen was collected during this field trip, comparison with the original description and the illustrations of the male terminalia provided by Londt (1983) suggested that the new specimens belong to an undescribed species. Further study revealed that the new species is more widespread and that several specimens previously identified as *Anasillomos chrysopos* also belong to the new species.

## Materials and methods

Morphological features were examined using a Zeiss SteREO Discovery.V12 stereo microscope. Wing length is measured from the tegula to the distal tip of the wing. The female genitalia and male terminalia were first excised and macerated in 10% potassium hydroxide (KOH) at 55°C followed by neutralization in acetic acid (CH_3_COOH) and rinsing in distilled water (H_2_O). They were temporarily stored in 75% ethanol (C_2_H_5_OH) for examination and illustration and eventually sealed in polyethylene vials containing 100% glycerine (C_3_H_8_O_3_) and attached to the specimen's pin.

### Terminology

Terminology follows Dikow (2009a) and Cumming and Wood (2009) (general morphology and abbreviations for setae), Stuckenberg (1999) (antennae), and Wootton and Ennos (1989) (wing venation). Abdominal tergites are abbreviated in the descriptions with 'T', and sternites are abbreviated with 'S'. The terms prothoracic, mesothoracic, and metathoracic are abbreviated 'pro', 'mes', and 'met', respectively. The term pubescence (adjective pubescent) refers to the short, fine microtrichia densely covering certain body parts. Other generalized terms follow the Torre-Bueno Glossary of Entomology by Nichols (1989).

### Species descriptions and re-descriptions

Species descriptions are based on composites of all specimens and not exclusively on the holotype and are compiled from a character matrix of 205 features and 242 character states assembled with Lucid Builder (version 3.5) and eventually exported as natural-language descriptions. These species descriptions have been deposited in the Zenodo data depository and can be accessed in XML-format following the SDD (Structure of Descriptive Data) standard (see also Suppl. material 1​). The structure of terminalia is only described once for the genus except when species differ. Additional species-specific features of the male terminalia should be interpreted from the provided photographs. All taxon names have been registered in ZooBank (Pyle and Michel 2008).

### Specimen occurrence data

The following data on species occurrences are given (where available): country, state/province, county, locality, geographic co-ordinates (formatted in both decimal and degrees minutes seconds latitude/longitude), elevation (in meters), date of collection (format: yyyy-mm-dd), habitat information, sampling protocol (if other than hand netting), collector, catalog number (a unique specimen number and any other identifying number), depository (institution and collection code), number of specimens, sex, life stage, name of person who identified the specimen, and any other previous identifications. Each specimen is listed with a unique specimen number (either an institutional catalog number or an AAM-XXXXXX number used by the author) that will allow the re-investigation as well as provide a unique Life Science Identifier (LSID). The occurrence of all species is illustrated in distribution maps plotted with SimpleMappr with all of those localities for which co-ordinates are available. Type localities are plotted with a square symbol while all other specimens are plotted with a circular symbol. The distribution map includes Biodiversity Hotspots *sensu* Conservation International. The specimen occurrence data are deposited as a Darwin Core Archive (DwC-A) in the Global Biodiversity Information Facility (GBIF) using the Integrated Publishing Toolkit (IPT) at the NMNH.

### Photographs

Whole habitus photographs of pinned specimens were taken using a Visionary Digital Passport II system (base and StackShot only), an Olympus E-30 digital SLR, a 50 mm macro lens (equivalent to 100 mm focal length in 35 mm photography), and a 25 mm extension tube. The specimens were illuminated by a Falcon FLDM-i200 LED dome-light for even and soft light. Photographs of the female and male terminalia were taken on a Zeiss SteREO Discovery.V12 stereo microscope at 50x magnification and an attached Olympus PEN E-PL5 digital camera. The dissected terminalia were placed in 75% ethanol in a glass dish and illuminated by a Schott VisiLED light source utilizing mixed bright-field (dorsal), dark-field (lateral), and transillumination (ventral). Adobe DNG-format images were stacked using HeliconFocus software. All photographs have been deposited in Morphbank:: Biological Imaging. These images will be automatically harvested by the Encyclopedia of Life (EOL) and are available under the respective species page.

### Keys

The dichotomous, interactive key has been build with Lucid Phoenix and the multi-access, matrix-based key with Lucid Builder and both have been deposited in the IdentifyLife archive, registered in Lucidcentral, and made available on the author's research web-site.

### Institutions providing specimens

Institutions providing specimens are listed below, together with the abbreviations used in the text when citing depositories (institutionCode), a link to the record in the Global Registry of Biodiversity Repositories (GRBio), and the people who kindly assisted: NMNW – National Museum of Namibia, Windhoek, Khomas, Namibia (A. Kirk-Spriggs); NMSA – KwaZulu-Natal Museum, Pietermaritzburg, KwaZulu-Natal, South Africa (B. Muller, J. Londt); SAMC – Iziko South African Museum, Cape Town, Western Cape, South Africa (D. Lawson, S. van Noort); USNM – National Museum of Natural History, Smithsonian Institution, Washington, DC, USA.

## Data resources

Morphbank: image collection ID 851000.

Zenodo: natural-language species descriptions from Lucid Builder in SDD format (also available as Suppl. material 1) – DOI 10.5281/zenodo.15790.

GBIF: specimen occurrence data-set – d61dc8a1-83f7-4905-8863-d19a5a89a0a4 – DOI 10.15468/bd6wxn.

**Figure 11.**
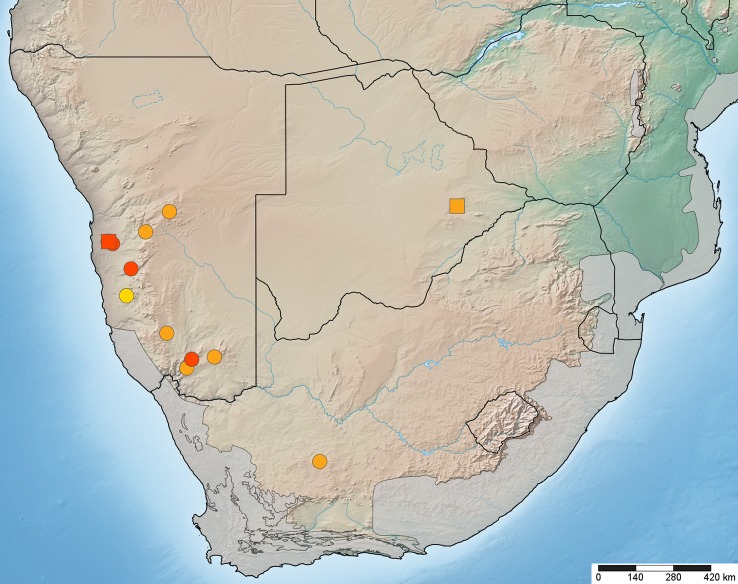
Map of southern Africa with elevational relief and biodiversity hotspots (in grey) showing distribution of *Anasillomos chrysopos* (orange) and *A. juergeni* sp. n. (red). Possible *A. juergeni* sp. n. from Awasib in yellow (see Discussion). Type localities with square symbol. Map data available in Google Earth KML file and also through GBIF (data-set d61dc8a1-83f7-4905-8863-d19a5a89a0a4).

SimpleMappr: distribution map – 2986 (as in Fig. 11) and in Google Earth as KML file.

Lucid Builder: illustrated, multi-entry, matrix-based identification key – asiloidflies.si.edu and IdentifyLife (also available as Suppl. material 2).

Lucid Phoenix: illustrated, dichotomous identification key – asiloidflies.si.edu.

## Taxon treatments

### Anasillomos

Londt, 1983

urn:lsid:zoobank.org:act:8C4E1B5F-2A3F-4B9E-92E2-AB8EC22DA046

Anasillomos Anasillomos chrysopos Londt, 1983Londt 1983: 284.

#### Diagnosis

*Anasillomos* has been included in the most recent key to Afrotropical Stenopogoninae *sensu* Papavero (1973) and Geller-Grimm (2004) with setose anatergites by Londt (2012). It can easily be distinguished from other southern African assassin-fly genera by the presence of strong macrosetae on the antepronotum, lateral postpronotum, and postpronotal lobes.

#### Taxon discussion

The phylogenetic studies on Asilidae based on both morphological (Dikow 2009a) and combined morphological and molecular data (Dikow 2009b) did not include representatives of *Anasillomos* and therefore the taxon cannot be directly placed in the phylogenetic classification proposed in Dikow (2009a). The external morphology suggests that *Anasillomos* belongs to the Stenopogoninae *sensu* Dikow (2009a) based on similarities to *Daspletis* Loew, 1859 and *Microstylum* Macquart, 1838 such as the male terminalia with a medio-distally projecting hypandrium (also known in other southern African genera though). These two genera have been included in Stenopogoninae: Enigmomorphini by Dikow (2009a). However, only the inclusion of *Anasillomos* specimens in a cladistic analysis can place this genus phylogenetically as overall similarity does not necessarily provide evidence as to the evolutionary relationships.

### Anasillomos chrysopos

Londt, 1983

urn:lsid:zoobank.org:act:38457B23-1147-4C3D-8263-6E93473A0156

Anasillomos chrysopos Londt, 1983: 284.

#### Materials

- **Type status:** Holotype. **Occurrence:** catalogNumber: NMSA-DIP-06957; recordedBy: Forchhammer, P.; individualCount: 1; sex: male; lifeStage: adult; **Taxon:** scientificNameID: urn:lsid:zoobank.org:act:38457B23-1147-4C3D-8263-6E93473A0156; scientificName: *Anasillomos chrysopos* Londt, 1983; family: Asilidae; genus: Anasillomos; specificEpithet: chrysopos; scientificNameAuthorship: Londt, 1983; **Location:** country: Botswana; stateProvince: Central; locality: Serowe; verbatimCoordinates: 22°23'00"S 026°43'00"E; decimalLatitude: -22.38333; decimalLongitude: 26.71667; **Identification:** identifiedBy: Londt, J.; dateIdentified: 1983; **Event:** eventDate: 1982-08-29; **Record Level:** institutionCode: NMSA; basisOfRecord: PreservedSpecimen
- **Type status:** Paratype. **Occurrence:** catalogNumber: NMSA-DIP-66448; recordedBy: Forchhammer, P.; individualCount: 1; sex: male; lifeStage: adult; **Taxon:** scientificNameID: urn:lsid:zoobank.org:act:38457B23-1147-4C3D-8263-6E93473A0156; scientificName: *Anasillomos chrysopos* Londt, 1983; family: Asilidae; genus: Anasillomos; specificEpithet: chrysopos; scientificNameAuthorship: Londt, 1983; **Location:** country: Botswana; stateProvince: Central; locality: Serowe; verbatimCoordinates: 22°23'00"S 026°43'00"E; decimalLatitude: -22.38333; decimalLongitude: 26.71667; **Identification:** identifiedBy: Londt, J.; dateIdentified: 1983; **Event:** eventDate: 1982-08-29; **Record Level:** institutionCode: NMSA; basisOfRecord: PreservedSpecimen
- **Type status:** Paratype. **Occurrence:** catalogNumber: NMSA-DIP-66449; recordedBy: Forchhammer, P.; individualCount: 1; sex: male; lifeStage: adult; **Taxon:** scientificNameID: urn:lsid:zoobank.org:act:38457B23-1147-4C3D-8263-6E93473A0156; scientificName: *Anasillomos chrysopos* Londt, 1983; family: Asilidae; genus: Anasillomos; specificEpithet: chrysopos; scientificNameAuthorship: Londt, 1983; **Location:** country: Botswana; stateProvince: Central; locality: Serowe; verbatimCoordinates: 22°23'00"S 026°43'00"E; decimalLatitude: -22.38333; decimalLongitude: 26.71667; **Identification:** identifiedBy: Londt, J.; dateIdentified: 1983; **Event:** eventDate: 1982-08-29; **Record Level:** institutionCode: NMSA; basisOfRecord: PreservedSpecimen
- **Type status:** Paratype. **Occurrence:** catalogNumber: NMSA-DIP-66450; individualCount: 1; sex: male; lifeStage: adult; **Taxon:** scientificNameID: urn:lsid:zoobank.org:act:38457B23-1147-4C3D-8263-6E93473A0156; scientificName: *Anasillomos chrysopos* Londt, 1983; family: Asilidae; genus: Anasillomos; specificEpithet: chrysopos; scientificNameAuthorship: Londt, 1983; **Location:** country: Namibia; stateProvince: Karas; county: Bethanie; locality: Riverside No. 135; verbatimCoordinates: 26°38'29"S 016°59'50"E; decimalLatitude: -26.64139; decimalLongitude: 16.99722; **Identification:** identifiedBy: Londt, J.; dateIdentified: 1983; **Event:** eventDate: 1971-10-23–1971-10-26; habitat: hover near ground among small shrubs; **Record Level:** institutionCode: NMSA; basisOfRecord: PreservedSpecimen
- **Type status:** Paratype. **Occurrence:** catalogNumber: NMSA-DIP-06963; individualCount: 1; sex: male; lifeStage: adult; **Taxon:** scientificNameID: urn:lsid:zoobank.org:act:38457B23-1147-4C3D-8263-6E93473A0156; scientificName: *Anasillomos chrysopos* Londt, 1983; family: Asilidae; genus: Anasillomos; specificEpithet: chrysopos; scientificNameAuthorship: Londt, 1983; **Location:** country: Namibia; stateProvince: Karas; county: Bethanie; locality: Riverside No. 135; verbatimCoordinates: 26°38'29"S 016°59'50"E; decimalLatitude: -26.64139; decimalLongitude: 16.99722; **Identification:** identifiedBy: Londt, J.; dateIdentified: 1983; **Event:** eventDate: 1971-10-23–1971-10-16; habitat: hover near ground among small shrubs; **Record Level:** institutionCode: NMSA; basisOfRecord: PreservedSpecimen
- **Type status:** Other material. **Occurrence:** catalogNumber: NMSA-DIP-66452; recordedBy: Dikow, T.; individualCount: 1; sex: female; lifeStage: adult; previousIdentifications: Anasillomos sp. by Londt, J.; Dikow, T. in 2000; **Taxon:** scientificNameID: urn:lsid:zoobank.org:act:38457B23-1147-4C3D-8263-6E93473A0156; scientificName: *Anasillomos chrysopos* Londt, 1983; family: Asilidae; genus: Anasillomos; specificEpithet: chrysopos; scientificNameAuthorship: Londt, 1983; **Location:** country: Namibia; stateProvince: Karas; locality: Ai-Ais Nature Reserve, NW Grünau (road D601); verbatimCoordinates: 27°30'45"S 017°50'01"E; decimalLatitude: -27.5125; decimalLongitude: 17.83361; **Identification:** identifiedBy: Dikow, T.; dateIdentified: 2014; **Event:** eventDate: 1999-12-09; **Record Level:** institutionCode: NMSA; basisOfRecord: PreservedSpecimen
- **Type status:** Other material. **Occurrence:** catalogNumber: NMSA-DIP-66451; recordedBy: Dikow, T.; individualCount: 1; sex: female; lifeStage: adult; previousIdentifications: Anasillomos sp. by Londt, J.; Dikow, T. in 2000; **Taxon:** scientificNameID: urn:lsid:zoobank.org:act:38457B23-1147-4C3D-8263-6E93473A0156; scientificName: *Anasillomos chrysopos* Londt, 1983; family: Asilidae; genus: Anasillomos; specificEpithet: chrysopos; scientificNameAuthorship: Londt, 1983; **Location:** country: Namibia; stateProvince: Karas; locality: Ai-Ais Nature Reserve, NW Grünau (road D601); verbatimCoordinates: 27°30'45"S 017°50'01"E; decimalLatitude: -27.5125; decimalLongitude: 17.83361; **Identification:** identifiedBy: Dikow, T.; dateIdentified: 2014; **Event:** eventDate: 1999-12-09; **Record Level:** institutionCode: NMSA; basisOfRecord: PreservedSpecimen
- **Type status:** Other material. **Occurrence:** catalogNumber: NMSA-DIP-06965; recordedBy: Londt, J.; individualCount: 1; sex: female; lifeStage: adult; **Taxon:** scientificNameID: urn:lsid:zoobank.org:act:38457B23-1147-4C3D-8263-6E93473A0156; scientificName: *Anasillomos chrysopos* Londt, 1983; family: Asilidae; genus: Anasillomos; specificEpithet: chrysopos; scientificNameAuthorship: Londt, 1983; **Location:** country: South Africa; stateProvince: Northern Cape; locality: Carnarvon, 2 km NE; verbatimElevation: 1350 m; verbatimCoordinates: 30°56'07"S 022°07'03"E; decimalLatitude: -30.93528; decimalLongitude: 22.1175; **Identification:** identifiedBy: Dikow, T.; dateIdentified: 2000; **Event:** eventDate: 1986-11-14; habitat: flat scrubland; **Record Level:** institutionCode: NMSA; basisOfRecord: PreservedSpecimen
- **Type status:** Other material. **Occurrence:** catalogNumber: INHS-33959; recordedBy: Irwin, M.; individualCount: 1; sex: female; lifeStage: adult; **Taxon:** scientificNameID: urn:lsid:zoobank.org:act:38457B23-1147-4C3D-8263-6E93473A0156; scientificName: *Anasillomos chrysopos* Londt, 1983; family: Asilidae; genus: Anasillomos; specificEpithet: chrysopos; scientificNameAuthorship: Londt, 1983; **Location:** country: Namibia; stateProvince: Karas; county: Karasburg; locality: Ai Ais, 30 km NE, Altdorn No. 3; verbatimElevation: 640 m; verbatimCoordinates: 27°48'16"S 017°40'02"E; decimalLatitude: -27.80444; decimalLongitude: 17.66722; **Identification:** identifiedBy: Dikow, T.; dateIdentified: 2014; **Event:** samplingProtocol: Malaise trap; eventDate: 1996-11-19; habitat: dry wash; **Record Level:** institutionCode: INHS; basisOfRecord: PreservedSpecimen
- **Type status:** Other material. **Occurrence:** individualCount: 1; sex: male; lifeStage: adult; **Taxon:** scientificNameID: urn:lsid:zoobank.org:act:38457B23-1147-4C3D-8263-6E93473A0156; scientificName: *Anasillomos chrysopos* Londt, 1983; family: Asilidae; genus: Anasillomos; specificEpithet: chrysopos; scientificNameAuthorship: Londt, 1983; **Location:** country: Namibia; stateProvince: Karas; locality: Awasib; verbatimCoordinates: 25°23'00"S 015°39'00"E; decimalLatitude: -25.38333; decimalLongitude: 15.65; **Identification:** identifiedBy: Londt, J.; dateIdentified: 1983; **Event:** eventDate: 1971-11-09–1971-11-10; **Record Level:** institutionCode: NMNW; basisOfRecord: PreservedSpecimen
- **Type status:** Other material. **Occurrence:** catalogNumber: NMNW-H60955; recordedBy: Irish, J.; individualCount: 1; sex: female; lifeStage: adult; **Taxon:** scientificNameID: urn:lsid:zoobank.org:act:38457B23-1147-4C3D-8263-6E93473A0156; scientificName: *Anasillomos chrysopos* Londt, 1983; family: Asilidae; genus: Anasillomos; specificEpithet: chrysopos; scientificNameAuthorship: Londt, 1983; **Location:** country: Namibia; stateProvince: Khomas; locality: Windhoek; verbatimCoordinates: 22°34'00"S 017°05'00"E; decimalLatitude: -22.56667; decimalLongitude: 17.08333; **Identification:** identifiedBy: Londt, J.; dateIdentified: 2005; **Event:** eventDate: 1984-09-26; **Record Level:** institutionCode: NMNW; basisOfRecord: PreservedSpecimen
- **Type status:** Other material. **Occurrence:** individualCount: 1; sex: female; lifeStage: adult; **Taxon:** scientificNameID: urn:lsid:zoobank.org:act:38457B23-1147-4C3D-8263-6E93473A0156; scientificName: *Anasillomos chrysopos* Londt, 1983; family: Asilidae; genus: Anasillomos; specificEpithet: chrysopos; scientificNameAuthorship: Londt, 1983; **Location:** country: Namibia; stateProvince: Khomas; locality: Windhoek; verbatimCoordinates: 22°34'00"S 017°05'00"E; decimalLatitude: -22.56667; decimalLongitude: 17.08333; **Identification:** identifiedBy: Londt, J.; Dikow, T.; dateIdentified: 2000; **Event:** eventDate: 1973-10-07–1973-10-12; **Record Level:** institutionCode: NMNW; basisOfRecord: PreservedSpecimen
- **Type status:** Other material. **Occurrence:** individualCount: 1; sex: undetermined; lifeStage: adult; **Taxon:** scientificNameID: urn:lsid:zoobank.org:act:38457B23-1147-4C3D-8263-6E93473A0156; scientificName: *Anasillomos chrysopos* Londt, 1983; family: Asilidae; genus: Anasillomos; specificEpithet: chrysopos; scientificNameAuthorship: Londt, 1983; **Location:** country: Namibia; stateProvince: Khomas; locality: Windhoek; verbatimCoordinates: 22°34'00"S 017°05'00"E; decimalLatitude: -22.56667; decimalLongitude: 17.08333; **Identification:** identifiedBy: Londt, J.; Dikow, T.; dateIdentified: 2000; **Event:** eventDate: 1973-10-07–1973-10-12; **Record Level:** institutionCode: NMNW; basisOfRecord: PreservedSpecimen
- **Type status:** Other material. **Occurrence:** individualCount: 1; sex: female; lifeStage: adult; **Taxon:** scientificNameID: urn:lsid:zoobank.org:act:38457B23-1147-4C3D-8263-6E93473A0156; scientificName: *Anasillomos chrysopos* Londt, 1983; family: Asilidae; genus: Anasillomos; specificEpithet: chrysopos; scientificNameAuthorship: Londt, 1983; **Location:** country: Namibia; stateProvince: Khomas; locality: Windhoek; verbatimCoordinates: 22°34'00"S 017°05'00"E; decimalLatitude: -22.56667; decimalLongitude: 17.08333; **Identification:** identifiedBy: Londt, J.; Dikow, T.; dateIdentified: 2000; **Event:** eventDate: 1973-10-04–1973-10-08; **Record Level:** institutionCode: NMNW; basisOfRecord: PreservedSpecimen
- **Type status:** Other material. **Occurrence:** catalogNumber: SAM-DIP-A008264; recordedBy: SAM Museum Staff; individualCount: 1; sex: male; lifeStage: adult; **Taxon:** scientificNameID: urn:lsid:zoobank.org:act:38457B23-1147-4C3D-8263-6E93473A0156; scientificName: *Anasillomos chrysopos* Londt, 1983; family: Asilidae; genus: Anasillomos; specificEpithet: chrysopos; scientificNameAuthorship: Londt, 1983; **Location:** country: Namibia; stateProvince: Karas; locality: Great Karas Mountains; verbatimCoordinates: 27°26'00"S 018°35'22"E; decimalLatitude: -27.43333; decimalLongitude: 18.58944; **Identification:** identifiedBy: Londt, J.; **Event:** eventDate: 1936-11-00; **Record Level:** institutionCode: SAMC; basisOfRecord: PreservedSpecimen
- **Type status:** Other material. **Occurrence:** catalogNumber: USNMENT00995574; recordedBy: Moore, A.; individualCount: 1; sex: female; lifeStage: adult; previousIdentifications: Anasillomos sp. by Londt, J. in 1994; **Taxon:** scientificNameID: urn:lsid:zoobank.org:act:38457B23-1147-4C3D-8263-6E93473A0156; scientificName: *Anasillomos chrysopos* Londt, 1983; family: Asilidae; genus: Anasillomos; specificEpithet: chrysopos; scientificNameAuthorship: Londt, 1983; **Location:** country: Namibia; stateProvince: Khomas; locality: Hakos Mountains, 191 km E Walvis Bay; verbatimCoordinates: 23°14'43"S 016°17'22"E; decimalLatitude: -23.24528; decimalLongitude: 16.28944; **Identification:** identifiedBy: Dikow, T.; dateIdentified: 2014; **Event:** eventDate: 1983-11-13; **Record Level:** institutionCode: USNM; basisOfRecord: PreservedSpecimen

#### Description

**Head:** wider than high, black; vertex slightly depressed (less than 60° angle on median margin of compound eye); facial swelling extending over lower ¾ of face, white pubescent; mystax white macrosetose on ventral margin and light brown macrosetose elsewhere, extending over lower ¾ of face; ommatidia of different size, at least some median ommatidia distinctly larger; postgena posterior margin simple, smooth; frons (at level of antennal insertion) more or less parallel-sided, apubescent antero-medially, grey to golden pubescent otherwise, white setose and light brown macrosetose laterally; ocellar tubercle golden pubescent, light brown setose and macrosetose; vertex golden pubescent, asetose; median occipital sclerite (m ocp scl) with several light brown macrosetae; postocular (pocl) setae straight, light brown macrosetae; occiput dorso-medially golden pubescent and laterally grey pubescent, dorso-medially with V-shaped apubescent stripes, white setose.

**Proboscis and maxillary palpus:** proboscis straight, black; postmentum plate-like, straight, ventral margin entirely smooth, white setose ventrally; prementum circular in cross section proximally, with dorso-median flange, white setose proximo-ventrally; labella reduced, fused to prementum entirely, small, only forming distal tip of proboscis, apex rounded; maxillary palpus two-segmented, black, distal palpomere cylindrical, yellowish setose and macrosetose; stipites fused entirely medially, apubescent, long white setose.

**Antenna:** light brown, lightly grey pubescent; scape 2x as long as pedicel, short white and light brown setose dorsally and long light brown macrosetose ventrally; pedicel white and light brown setose distally; postpedicel cylindrical (same diameter throughout), 1.5x as long as scape and pedicel combined, sparsely white setose dorso-distally; stylus comprised of 1 element, asetose; apical seta-like sensory element situated apically on stylus.

**Thorax:** brown to dark brown, postpronotal lobes and lateral scutum light brown to orange; prosternum apubescent, separated from proepisternum, square to rectangular in shape (straight dorsally); proepisternum grey pubescent, white setose and light brown macrosetose; cervical sclerite long white setose; antepronotum golden pubescent, white setose and light brown macrosetose; postpronotum golden pubescent, white setose and light brown macrosetose, postpronotal lobe white setose and long light brown macrosetose; pleuron grey and golden pubescent; proepimeron white setose; anepisternum anterior half asetose, dorso-medially white to light brown macrosetose (6–7 macrosetae), posterior half sparsely long white setose, supero-posteriorly white setose (not macrosetose); anterior basalare asetose, posterior basalare asetose; anepimeron predominantly asetose, antero-dorsally white setose, katepisternum predominantly asetose, postero-dorsally white setose, katepimeron asetose, katatergite white setose and long yellowish macrosetose; meron + metanepisternum asetose, metakatepisternum asetose, metepimeron sparsely white setose; anatergite long white setose; scutum predominantly light brown pubescent, median stripe (extending beyond transverse suture) grey pubescent, paramedian stripes (reaching transverse suture) light brown pubescent, narrow grey pubescent stripes (merging with median stripe beyond transverse suture) grey pubescent, broad stripes (anteriorly wide and posteriorly narrow) brown pubescent (especially visible in anterior and lateral view), sub–lateral spots (1 anterior and 2 posterior to transverse suture) brown pubescent; scutum setation: short white setose anterior and long white setose posterior to transverse suture, setae with small sockets, 3 npl setae, 4 spa setae, 4 pal setae, 2 light brown presutural dc macrosetae and 3–4 light brown postsutural dc macrosetae, acr setae white, presuturally short and longer postsuturally, median posterior scutum (between dc setae) long white setose, setae directed posteriorly; scutellum grey pubescent, ds sctl setae present, long white setae on posterior margin, ap sctl setae present, 6–8 long light brown macrosetae; postmetacoxal area entirely membranous.

**Leg:** light brown to brown, apubescent, all setae circular in cross section; pro coxa brown, grey pubescent, white setose and light brown macrosetose; pro femur brown, dorsally dark brown, short white setose, light brown macrosetose: 1–2 antero-median, 8–12 postero-dorsal, and 4–5 ventral macrosetae; pro tibia light brown to brown, short white setose, yellowish macrosetose: 7 in 1 antero-dorsal row, 5 in 1 postero-dorsal row, 7 in 1 postero-ventral row, 3 long in 1 postero-ventral row, distally with 6–7 long white macrosetae; mes coxa brown, grey pubescent, white setose and yellowish macrosetose; mes femur brown, dorsally dark brown, short white setose, light brown macrosetose: 2 antero-median, 6–8 postero-dorsal (longest distally), and 5–6 ventral macrosetae; mes tibia brown, short white setose, yellowish macrosetose: 4 in 1 antero-dorsal row, 5 in 1 dorsal row, 5 in 1 posterior row, 3 in 1 postero-ventral row; met coxa brown, grey pubescent, white setose and light brown macrosetose, anteriorly without any protuberance; met trochanter white setose and yellowish macrosetose, cylindrical, medially without protuberance; met femur brown, dorsally dark brown, short white setose, light brown macrosetose: 6–8 anterior, 4–5 ventral, and 3 posterior macrosetae; met tibia brown, straight, short white setose, yellowish macrosetose: 3–4 in 1 antero-dorsal row, 3–4 in 1 dorsal row, 3 in 1 ventral row; proximal pro, mes, and met tarsomeres as long as following 2 tarsomeres combined, pro, mes, and met tarsomeres white setose dorsally, pro tarsomeres white macrosetose disto-laterally and disto-ventrally, mes and met tarsomeres white macrosetose laterally and ventrally; pulvilli well-developed (as long as claw); claw abruptly angled distally, pointed; empodium setiform, well-developed (as long as pulvilli).

**Wing:** length 8.1–9.5 mm, wing membrane hyaline, without microtrichia; C circumambient (developed around entire wing), anterior wing margin straight; R₂₊₃ distally distinctly arching anteriorly, r₁ open; R₄ terminating anterior to wing apex, distinctly arching anteriorly, stump vein (R₃) absent; r₄ open, R₄ and R₅ diverging from each other; R₅ not reaching C (or wing margin); r₅ closed and petiolate; M₁ not reaching C (or wing margin); cell d closed by base of M₂ and m-m, M₂ and m-m not aligned, r-m situated in distal half of cell d; m₃ closed at C (non-petiolate) or open, M₃ and M₄ approximating at C; cu*a* closed at C (non-petiolate); alula well-developed; microtrichia on posterior wing margin arranged in a single plane.

**Abdomen:** brown to black, laterally light brown to orange, tergites smooth, setae with small sockets only; T1 white setose, laterally long light brown macrosetose, grey pubescent, entirely sclerotized medially, dorsal surface smooth, without protuberances; T2–8 entirely sclerotized, predominantly black, sometimes brown to orange laterally, predominantly grey pubescent, T2 with apubescent stripe from antero-lateral corner to postero-median margin, T3–5 with narrower apubescent stripes as on T2, white setose, yellowish macrosetae postero-laterally on T2–4 (most pronounced on T2); marginal macrosetae present on T2–4, medial macrosetae present on T2; S1–8 black, lightly grey pubescent, white, erect setose.

**Female:** T7 and S7 without modifications, ovipositor comprised of 8th and following segments, T6–8 apubescent, setation directed anteriorly on T6–7 and erect on T8; T8 anteriorly with internal rectangular apodeme (entirely fused to T), S8 plate-like, hypogynial valves extending; T9 and T10 entirely fused, sclerites not distinguishable, T10 divided into two heavily sclerotized acanthophorite plates, with 6–7, yellowish acanthophorite spurs per plate; 3 spermathecae, all equally large, reaching anterior end of segment 6; common spermathecal duct short, not extending beyond tip of furca, individual spermathecal ducts long; ejection apparatus absent; spermathecal reservoirs formed by more or less expanded and coiled ducts, weakly sclerotized; furca (S9) formed by single, inverted V-shaped sclerite, median sclerite (at posterior tip) absent, anterior furcal apodeme absent.

**Male (Fig. 4a, b, c):** T1–T8 and S1–S8 entire (without modifications); hypopygium dark brown, rotated by 180°, directed posteriorly; epandrium divided medially into two halves, joined proximally; hypandrium well-developed, triangular, distally with short postero-median projection, distinctly separated from epandrium by gonocoxite, not fused to gonocoxite; gonocoxite partially fused to epandrium proximally, gonocoxal apodeme present, short, at most slightly extending hypopygium proximally; gonostylus present, positioned distally on gonocoxite; subepandrial sclerite asetose, ventrally with median, wavy longitudinal ridge, laterally straight (without protuberances), distally simple, straight margin; cerci separate (not fused medially); 1 aedeagal prong, tip pointed, without any protuberance, dorsal aedeagal sheath short, sperm sac entirely free; lateral ejaculatory process present, large triangular sclerite, free (not surrounded by ventral aedeagal sheath); ejaculatory apodeme formed by single vertical plate (two lateral surfaces).

#### Diagnosis

The species is distinguished from its congener by the dorso-medially white macrosetose anepisternum (in addition to white setae, Figs 2b, 3b), the distinct pubescence pattern on the lateral abdominal tergites (T2–5 with apubescent stripe from antero-lateral corner to postero-median margin, Fig. 3b), and the presence of long macrosetae on the tip of the pointed hypandrium (almost reaching tip of epandrium, Fig. 4b​).

#### Distribution

Known from Botswana, Namibia, and South Africa and therefore widely distributed in southern Africa, but rarely collected (Fig. 11).

#### Taxon discussion

The type locality of *A. chrysopos* lies in eastern Botswana, far removed from the majority of specimens collected in western Namibia. There is little morphological variation within the species except for the setation color, which can vary from light brown to yellow.

#### Type locality

Botswana: Central: Serowe (22°23'00"S 026°43'00"E).

#### Biodiversity hotspot

Not known to occur in any of the southern African biodiversity hotspots (Cape Floristic Region, Maputaland-Pondoland-Albany, or Succulent Karoo).

### Anasillomos juergeni

Dikow, 2014 sp. n.

urn:lsid:zoobank.org:act:1D180F02-AA54-4828-8D46-84566D07C173

#### Materials

- **Type status:** Holotype. **Occurrence:** catalogNumber: USNMENT00832201; recordedBy: Dikow, T.; individualCount: 1; sex: male; lifeStage: adult; **Taxon:** scientificNameID: urn:lsid:zoobank.org:pub:713C351C-971A-40F8-9368-FE39AFE266D7; scientificName: *Anasillomos juergeni* sp. n.; family: Asilidae; genus: Anasillomos; specificEpithet: juergeni; **Location:** country: Namibia; stateProvince: Erongo; locality: Namib-Skeleton Coast National Park, Gobabeb; verbatimElevation: 441 m; verbatimCoordinates: 23°34'17"S 015°02'52"E; decimalLatitude: -23.57139; decimalLongitude: 15.04778; **Identification:** identifiedBy: Dikow, T.; dateIdentified: 2014; **Event:** eventDate: 2012-02-07; habitat: on dune with grass boulders, perching on sand; **Record Level:** institutionCode: NMNW; basisOfRecord: PreservedSpecimen
- **Type status:** Paratype. **Occurrence:** catalogNumber: USNMENT00832190; recordedBy: Dikow, T.; individualCount: 1; sex: female; lifeStage: adult; **Taxon:** scientificNameID: urn:lsid:zoobank.org:pub:713C351C-971A-40F8-9368-FE39AFE266D7; scientificName: *Anasillomos juergeni* sp. n.; family: Asilidae; genus: Anasillomos; specificEpithet: juergeni; **Location:** country: Namibia; stateProvince: Erongo; locality: Namib-Skeleton Coast National Park, Gobabeb; verbatimElevation: 441 m; verbatimCoordinates: 23°34'17"S 015°02'52"E; decimalLatitude: -23.57139; decimalLongitude: 15.04778; **Identification:** identifiedBy: Dikow, T.; dateIdentified: 2014; **Event:** eventDate: 2012-02-07; habitat: on dune with grass boulders, perching on sand; **Record Level:** institutionCode: USNM; basisOfRecord: PreservedSpecimen
- **Type status:** Paratype. **Occurrence:** catalogNumber: USNMENT00832192; recordedBy: Dikow, T.; individualCount: 1; sex: female; lifeStage: adult; **Taxon:** scientificNameID: urn:lsid:zoobank.org:pub:713C351C-971A-40F8-9368-FE39AFE266D7; scientificName: *Anasillomos juergeni* sp. n.; family: Asilidae; genus: Anasillomos; specificEpithet: juergeni; **Location:** country: Namibia; stateProvince: Erongo; locality: Namib-Skeleton Coast National Park, Homeb; verbatimElevation: 445 m; verbatimCoordinates: 23°38'34"S 015°10'55"E; decimalLatitude: -23.64278; decimalLongitude: 15.18194; **Identification:** identifiedBy: Dikow, T.; dateIdentified: 2014; **Event:** eventDate: 2012-02-06; habitat: on dune, perching on sand; **Record Level:** institutionCode: USNM; basisOfRecord: PreservedSpecimen
- **Type status:** Other material. **Occurrence:** catalogNumber: NMSA-DIP-06951; recordedBy: Dikow, T.; individualCount: 1; sex: female; lifeStage: adult; previousIdentifications: Anasillomos sp. by Londt, J.; Dikow, T. in 2000; **Taxon:** scientificNameID: urn:lsid:zoobank.org:pub:713C351C-971A-40F8-9368-FE39AFE266D7; scientificName: *Anasillomos juergeni* sp. n.; family: Asilidae; genus: Anasillomos; specificEpithet: juergeni; **Location:** country: Namibia; stateProvince: Karas; locality: Ai-Ais Nature Reserve, NW Grünau (road D601); verbatimCoordinates: 27°30'45"S 017°50'01"E; decimalLatitude: -27.5125; decimalLongitude: 17.83361; **Identification:** identifiedBy: Dikow, T.; dateIdentified: 2014; **Event:** eventDate: 1999-12-09; **Record Level:** institutionCode: NMSA; basisOfRecord: PreservedSpecimen
- **Type status:** Other material. **Occurrence:** catalogNumber: NMSA-DIP-66453; recordedBy: Dikow, T.; individualCount: 1; sex: female; lifeStage: adult; previousIdentifications: Anasillomos sp. by Londt, J.; Dikow, T. in 2000; **Taxon:** scientificNameID: urn:lsid:zoobank.org:pub:713C351C-971A-40F8-9368-FE39AFE266D7; scientificName: *Anasillomos juergeni* sp. n.; family: Asilidae; genus: Anasillomos; specificEpithet: juergeni; **Location:** country: Namibia; stateProvince: Karas; locality: Ai-Ais Nature Reserve, NW Grünau (road D601); verbatimCoordinates: 27°30'45"S 017°50'01"E; decimalLatitude: -27.5125; decimalLongitude: 17.83361; **Identification:** identifiedBy: Dikow, T.; dateIdentified: 2014; **Event:** eventDate: 1999-12-09; **Record Level:** institutionCode: NMSA; basisOfRecord: PreservedSpecimen
- **Type status:** Other material. **Occurrence:** catalogNumber: NMSA-DIP-06970; individualCount: 1; sex: female; lifeStage: adult; previousIdentifications: Anasillomos chrysopos by Londt, J. in 1983; **Taxon:** scientificNameID: urn:lsid:zoobank.org:pub:713C351C-971A-40F8-9368-FE39AFE266D7; scientificName: *Anasillomos juergeni* sp. n.; family: Asilidae; genus: Anasillomos; specificEpithet: juergeni; taxonRemarks: Paratype of Anasillomos chrysopos Londt, 1983; **Location:** country: Namibia; stateProvince: Hardap; county: Maltahöhe; locality: Sesriem No. 137; verbatimCoordinates: 24°29'00"S 015°48'00"E; decimalLatitude: -24.48333; decimalLongitude: 15.8; **Identification:** identifiedBy: Dikow, T.; dateIdentified: 2014; **Event:** eventDate: 1973-02-15–1973-02-17; **Record Level:** institutionCode: NMSA; basisOfRecord: PreservedSpecimen
- **Type status:** Other material. **Occurrence:** catalogNumber: NMSA-DIP-06958; recordedBy: Cunningham, A.; individualCount: 1; sex: female; lifeStage: adult; previousIdentifications: Anasillomos chrysopos by Londt, J. in 1983; **Taxon:** scientificNameID: urn:lsid:zoobank.org:pub:713C351C-971A-40F8-9368-FE39AFE266D7; scientificName: *Anasillomos juergeni* sp. n.; family: Asilidae; genus: Anasillomos; specificEpithet: juergeni; taxonRemarks: Paratype of Anasillomos chrysopos Londt, 1983; **Location:** country: Namibia; stateProvince: Erongo; locality: Gobabeb, Kuiseb River; verbatimCoordinates: 23°33'37"S 015°02'00"E; decimalLatitude: -23.56028; decimalLongitude: 15.03333; **Identification:** identifiedBy: Dikow, T.; dateIdentified: 2014; **Event:** eventDate: 1976-10-07; habitat: on dry river bed; **Record Level:** institutionCode: NMSA; basisOfRecord: PreservedSpecimen
- **Type status:** Paratype. **Occurrence:** catalogNumber: USNMENT00832195; recordedBy: Dikow, T.; individualCount: 1; sex: female; lifeStage: adult; **Taxon:** scientificNameID: urn:lsid:zoobank.org:pub:713C351C-971A-40F8-9368-FE39AFE266D7; scientificName: *Anasillomos juergeni* sp. n.; family: Asilidae; genus: Anasillomos; specificEpithet: juergeni; **Location:** country: Namibia; stateProvince: Erongo; locality: Namib-Skeleton Coast National Park, Gobabeb; verbatimElevation: 393 m; verbatimCoordinates: 23°33'50"S 015°02'01"E; decimalLatitude: -23.56389; decimalLongitude: 15.03361; **Identification:** identifiedBy: Dikow, T.; dateIdentified: 2014; **Event:** eventDate: 2012-02-05; habitat: small vegetated dunes and adjacent grassy flats, perching on low dead vegetation; **Record Level:** institutionCode: USNM; basisOfRecord: PreservedSpecimen
- **Type status:** Paratype. **Occurrence:** catalogNumber: USNMENT00832196; recordedBy: Dikow, T.; individualCount: 1; sex: female; lifeStage: adult; **Taxon:** scientificNameID: urn:lsid:zoobank.org:pub:713C351C-971A-40F8-9368-FE39AFE266D7; scientificName: *Anasillomos juergeni* sp. n.; family: Asilidae; genus: Anasillomos; specificEpithet: juergeni; **Location:** country: Namibia; stateProvince: Erongo; locality: Namib-Skeleton Coast National Park, Gobabeb; verbatimElevation: 441 m; verbatimCoordinates: 23°34'17"S 015°02'52"E; decimalLatitude: -23.57139; decimalLongitude: 15.04778; **Identification:** identifiedBy: Dikow, T.; dateIdentified: 2014; **Event:** eventDate: 2012-02-07; habitat: on dune with grass boulders, perching on sand; **Record Level:** institutionCode: USNM; basisOfRecord: PreservedSpecimen
- **Type status:** Paratype. **Occurrence:** catalogNumber: USNMENT00832197; recordedBy: Dikow, T.; individualCount: 1; sex: female; lifeStage: adult; **Taxon:** scientificNameID: urn:lsid:zoobank.org:pub:713C351C-971A-40F8-9368-FE39AFE266D7; scientificName: *Anasillomos juergeni* sp. n.; family: Asilidae; genus: Anasillomos; specificEpithet: juergeni; **Location:** country: Namibia; stateProvince: Erongo; locality: Namib-Skeleton Coast National Park, Gobabeb; verbatimElevation: 393 m; verbatimCoordinates: 23°33'50"S 015°02'01"E; decimalLatitude: -23.56389; decimalLongitude: 15.03361; **Identification:** identifiedBy: Dikow, T.; dateIdentified: 2014; **Event:** eventDate: 2012-02-05; habitat: small vegetated dunes and adjacent grassy flats, perching on low dead vegetation; **Record Level:** institutionCode: NMNW; basisOfRecord: PreservedSpecimen
- **Type status:** Paratype. **Occurrence:** catalogNumber: USNMENT00832198; recordedBy: Dikow, T.; individualCount: 1; sex: female; lifeStage: adult; **Taxon:** scientificNameID: urn:lsid:zoobank.org:pub:713C351C-971A-40F8-9368-FE39AFE266D7; scientificName: *Anasillomos juergeni* sp. n.; family: Asilidae; genus: Anasillomos; specificEpithet: juergeni; **Location:** country: Namibia; stateProvince: Erongo; locality: Namib-Skeleton Coast National Park, Gobabeb; verbatimElevation: 441 m; verbatimCoordinates: 23°34'17"S 015°02'52"E; decimalLatitude: -23.57139; decimalLongitude: 15.04778; **Identification:** identifiedBy: Dikow, T.; dateIdentified: 2014; **Event:** eventDate: 2012-02-07; habitat: on dune with grass boulders, perching on sand; **Record Level:** institutionCode: USNM; basisOfRecord: PreservedSpecimen
- **Type status:** Paratype. **Occurrence:** catalogNumber: USNMENT00832200; recordedBy: Dikow, T.; individualCount: 1; sex: female; lifeStage: adult; **Taxon:** scientificNameID: urn:lsid:zoobank.org:pub:713C351C-971A-40F8-9368-FE39AFE266D7; scientificName: *Anasillomos juergeni* sp. n.; family: Asilidae; genus: Anasillomos; specificEpithet: juergeni; **Location:** country: Namibia; stateProvince: Erongo; locality: Namib-Skeleton Coast National Park, Gobabeb; verbatimElevation: 441 m; verbatimCoordinates: 23°34'17"S 015°02'52"E; decimalLatitude: -23.57139; decimalLongitude: 15.04778; **Identification:** identifiedBy: Dikow, T.; dateIdentified: 2014; **Event:** eventDate: 2012-02-07; habitat: on dune with grass boulders, perching on sand; **Record Level:** institutionCode: NMNW; basisOfRecord: PreservedSpecimen
- **Type status:** Paratype. **Occurrence:** catalogNumber: USNMENT00832202; recordedBy: Dikow, T.; individualCount: 1; sex: female; lifeStage: adult; **Taxon:** scientificNameID: urn:lsid:zoobank.org:pub:713C351C-971A-40F8-9368-FE39AFE266D7; scientificName: *Anasillomos juergeni* sp. n.; family: Asilidae; genus: Anasillomos; specificEpithet: juergeni; **Location:** country: Namibia; stateProvince: Erongo; locality: Namib-Skeleton Coast National Park, Gobabeb; verbatimElevation: 441 m; verbatimCoordinates: 23°34'17"S 015°02'52"E; decimalLatitude: -23.57139; decimalLongitude: 15.04778; **Identification:** identifiedBy: Dikow, T.; dateIdentified: 2014; **Event:** eventDate: 2012-02-07; habitat: on dune with grass boulders, perching on sand; **Record Level:** institutionCode: USNM; basisOfRecord: PreservedSpecimen
- **Type status:** Other material. **Occurrence:** catalogNumber: USNMENT00914550; recordedBy: Dikow, T.; individualCount: 1; sex: undetermined; lifeStage: adult; **Taxon:** scientificNameID: urn:lsid:zoobank.org:pub:713C351C-971A-40F8-9368-FE39AFE266D7; scientificName: *Anasillomos juergeni* sp. n.; family: Asilidae; genus: Anasillomos; specificEpithet: juergeni; **Location:** country: Namibia; stateProvince: Erongo; locality: Namib-Skeleton Coast National Park, Gobabeb; verbatimElevation: 441 m; verbatimCoordinates: 23°34'17"S 015°02'52"E; decimalLatitude: -23.57139; decimalLongitude: 15.04778; **Identification:** identifiedBy: Dikow, T.; dateIdentified: 2014; **Event:** eventDate: 2012-02-07; habitat: on dune with grass boulders, perching on sand; **Record Level:** institutionCode: USNM; basisOfRecord: PreservedSpecimen
- **Type status:** Other material. **Occurrence:** catalogNumber: USNMENT00995573; recordedBy: Moore, A.; individualCount: 1; sex: male; lifeStage: adult; previousIdentifications: Anasillomos sp. by Londt, J. in 1994; **Taxon:** scientificNameID: urn:lsid:zoobank.org:pub:713C351C-971A-40F8-9368-FE39AFE266D7; scientificName: *Anasillomos juergeni* sp. n.; family: Asilidae; genus: Anasillomos; specificEpithet: juergeni; **Location:** country: Namibia; stateProvince: Erongo; locality: Gobabeb, Namib Desert Research Station; verbatimCoordinates: 23°33'37"S 015°02'26"E; decimalLatitude: -23.56028; decimalLongitude: 15.04056; **Identification:** identifiedBy: Dikow, T.; dateIdentified: 2014; **Event:** eventDate: 1983-11-17; **Record Level:** institutionCode: USNM; basisOfRecord: PreservedSpecimen

#### Description

**Head:** wider than high, black; vertex slightly depressed (less than 60° angle on median margin of compound eye); facial swelling extending over lower ¾ of face, white pubescent; mystax white macrosetose, extending over lower ¾ of face; ommatidia of different size, at least some median ommatidia distinctly larger; postgena posterior margin simple, smooth; frons (at level of antennal insertion) more or less parallel-sided, apubescent antero-medially, grey to golden pubescent otherwise, white setose and macrosetose laterally; ocellar tubercle golden pubescent, white setose and macrosetose; vertex golden pubescent, asetose; median occipital sclerite (m ocp scl) with several white macrosetae; postocular (pocl) setae straight, white macrosetose; occiput predominantly grey pubescent, dorso-medially with V-shaped apubescent stripes and medially with longitudinal light brown pubescent stripe, white setose.

**Proboscis and maxillary palpus:** proboscis straight, black; postmentum plate-like, straight, ventral margin entirely smooth, white setose ventrally; prementum circular in cross section proximally, with dorso-median flange, white setose proximo-ventrally; labella reduced, fused to prementum entirely, small, only forming distal tip of proboscis, apex rounded; maxillary palpus two-segmented, black, distal palpomere cylindrical, yellowish setose, distally white macrosetose; stipites fused entirely medially, apubescent, long white setose.

**Antenna:** light brown, lightly grey pubescent; scape 2x as long as pedicel, short white setose dorsally and long white macrosetose ventrally; pedicel white setose distally; postpedicel cylindrical (same diameter throughout), almost 2x as long as scape and pedicel combined, sparsely white setose dorso-distally; stylus comprised of 1 element, asetose; apical seta-like sensory element situated apically on stylus.

**Thorax:** dark brown to black, postpronotal lobes light brown to orange; prosternum apubescent, separated from proepisternum, square to rectangular in shape (straight dorsally); proepisternum grey pubescent, white setose and macrosetose; cervical sclerite long white setose; antepronotum golden pubescent, white setose and macrosetae; postpronotum golden pubescent, medially white setose, laterally long yellowish macrosetose, postpronotal lobe white setose and long yellowish macrosetose; pleuron predominantly grey and golden pubescent, katepimeron and antero-dorsal meron + metanepisternum apubescent; proepimeron white setose; anepisternum anterior half asetose, posterior half long white setose, supero-posteriorly white setose (not macrosetose); anterior basalare asetose, posterior basalare asetose; anepimeron predominantly asetose, antero-dorsally white setose, katepisternum predominantly asetose, postero-dorsally white setose, katepimeron asetose, katatergite white setose and long yellowish macrosetose; meron + metanepisternum asetose, metakatepisternum asetose, metepimeron sparsely white setose; anatergite long white setose; scutum predominantly grey pubescent, narrow paramedian stripes (just reaching past transverse suture) and sub–lateral spots (1 anterior and 2 posterior to transverse suture) brown pubescent; scutum setation: short white setose anterior and long white setose posterior to transverse suture, setae with small sockets, 3 npl setae, 3–4 spa setae, 4 pal setae, 1 white presutural dc macroseta and 3 white postsutural dc macrosetae, acr setae white, presuturally short and longer postsuturally, median posterior scutum (between dc setae) long white setose, setae directed posteriorly; scutellum grey pubescent, ds sctl setae present, long white setae in posterior half, ap sctl setae present, 5–6 long white macrosetae; postmetacoxal area entirely membranous.

**Leg:** light brown to brown, apubescent, all setae circular in cross section; pro coxa dark brown, grey pubescent, white setose and macrosetose; pro femur brown, dorsally dark brown, short white setose, white macrosetose: 1 antero-median, 5–6 postero-dorsal, and 6–7 ventral macrosetae; pro tibia brown, short white setose, white macrosetose: 5 in 1 antero-dorsal row, 5 in 1 postero-dorsal row, 4 long in 1 postero-ventral row, distally with 6–7 long white macrosetae; mes coxa dark brown, grey pubescent, white setose and macrosetose; mes femur brown, dorsally dark brown, short white setose, white macrosetose: 2 antero-median, 6–7 postero-dorsal, and 5–6 ventral macrosetae; mes tibia brown, short white setose, white macrosetose: 3 in 1 antero-dorsal row, 2 in 1 antero-ventral row, 4 in 1 dorsal row, 3 in 1 postero-ventral row; met coxa brown, grey pubescent, white setose and macrosetose, anteriorly without any protuberance; met trochanter white setose and macrosetose, cylindrical, medially without protuberance; met femur brown, dorsally dark brown, short white setose, white macrosetose: 6 anterior, 6–7 ventral, and 4–5 postero-ventral macrosetae; met tibia brown, straight, short white setose, white macrosetose: 4 in 1 antero-dorsal row, 3 in 1 dorsal row, 4–5 in 1 ventral row; proximal pro, mes, and met tarsomeres as long as following 2 tarsomeres combined, pro, mes, and met tarsomeres white setose dorsally, pro tarsomeres white macrosetose disto-laterally and disto-ventrally, mes and met tarsomeres white macrosetose laterally and ventrally; pulvilli well-developed (as long as claw); claw abruptly angled distally, pointed; empodium setiform, well-developed (as long as pulvilli).

**Wing (Fig. 5d):** length 8.0–9.5 mm, wing membrane hyaline, narrowly colored light brown along distal veins, without microtrichia; C circumambient (developed around entire wing), anterior wing margin straight; R₂₊₃ distally distinctly arching anteriorly, r₁ open; R₄ terminating anterior to wing apex, distinctly arching anteriorly, stump vein (R₃) absent; r₄ open, R₄ and R₅ diverging from each other; R₅ not reaching C (or wing margin); r₅ closed and petiolate; M₁ not reaching C (or wing margin); cell d closed by base of M₂ and m-m, M₂ and m-m not aligned, r-m situated in distal half of cell d; m₃ open, M₃ and M₄ approximating at C; cu*a* closed at C (non-petiolate); alula well-developed; microtrichia on posterior wing margin arranged in a single plane.

**Abdomen:** brown to black, laterally light brown to orange, tergites smooth, setae with small sockets only; T1 white setose, laterally long white macrosetose, grey pubescent, entirely sclerotized medially, dorsal surface smooth, without protuberances; T2–8 entirely sclerotized, predominantly black, sometimes brown to orange laterally, lightly grey pubescent, medially with distinct longitudinal dark grey pubescent stripe, white setose, setae longest on T2, medial setae directed laterally; marginal macrosetae absent from T2–8, medial macrosetae absent from T2–8; S1–8 black, lightly grey pubescent, white, erect setose.

**Female (Fig. 9a, b, c):** T7 and S7 without modifications, ovipositor comprised of 8th and following segments, T6–8 apubescent, setation directed anteriorly on T6–7 and erect on T8; T8 anteriorly with internal rectangular apodeme (entirely fused to T), S8 plate-like, hypogynial valves extending; T9 and T10 entirely fused, sclerites not distinguishable, T10 divided into two heavily sclerotized acanthophorite plates, with 7, white acanthophorite spurs per plate; 3 spermathecae, all equally large, reaching anterior end of segment 6; common spermathecal duct short, not extending beyond tip of furca, individual spermathecal ducts long; ejection apparatus absent; spermathecal reservoirs formed by more or less expanded and coiled ducts, weakly sclerotized; furca (S9) formed by single, inverted V-shaped sclerite, median sclerite (at posterior tip) absent, anterior furcal apodeme absent.

**Male (Fig. 8a, b, c):** T1–T8 and S1–S8 entire (without modifications); hypopygium dark brown, rotated by 180°, directed posteriorly; epandrium divided medially into two halves, joined proximally; hypandrium well-developed, triangular, distally with short postero-median projection, distinctly separated from epandrium by gonocoxite, not fused to gonocoxite; gonocoxite partially fused to epandrium proximally, gonocoxal apodeme present, short, at most slightly extending hypopygium proximally; gonostylus present, positioned distally on gonocoxite; subepandrial sclerite asetose, ventrally smooth (without protuberances), laterally straight (without protuberances), distally simple, straight margin; cerci separate (not fused medially); 1 aedeagal prong, tip pointed, with dorsal bipartite protuberance, dorsal aedeagal sheath short, sperm sac entirely free; lateral ejaculatory process present, large triangular sclerite, free (not surrounded by ventral aedeagal sheath); ejaculatory apodeme formed by single vertical plate (two lateral surfaces).

#### Diagnosis

The species is distinguished from its congener by the dorso-posterior white setose anepisternum (not macrosetose, Figs 5b, 6b, 7b), the distinct median longitudinal dark grey pubescent stripe on the abdominal tergites (Figs 5a, 6a), the absence of a pubescence pattern on the lateral abdominal tergites (Figs 5b, 7b), the presence of a dorsal bipartite protuberance on the aedeagus tip (Fig. 8c), and the presence of only short macrosetae on the tip of the pointed hypandrium (Fig. 8b​).

#### Etymology

The species is named after and dedicated to the memory of my late father, Jürgen Dikow, who has always been supportive of my entomological work and was excited to hear about every new species discovery I made.

#### Distribution

The species is so far only known from Namibia and in particular from the eastern edge of the Namib desert sand dunes (Gobabeb, Homeb, and Sesriem) as well as the Karoo in southern Namibia (near Grünau) (Fig. 11).

#### Biology

All recently collected specimens during field work at or near the Gobabeb Research and Training Center conducted in February 2012 were perching on sand. The majority of specimens were collected on the large sand dunes south of the station and Kuiseb river bed (Fig. 10a, b) while a few were encountered on the small dunes west of the Kuiseb river bed (Fig. 10c). The flies are very fast fliers and fly away even when one is several meters away. An attempt was made to photograph them in the field, but I was unable to get sufficiently close (see Fig. 10a with a fly in the center).

Four female specimens have been captured with prey at or near Gobabeb of which three were feeding on Bombyliidae (Diptera) and one on Ichneumonidae (Hymenoptera).

#### Type locality

Namibia: Erongo: Namib-Skeleton Coast National Park, Gobabeb (dunes south of station at 23°34'17"S 015°02'52"E).

#### Biodiversity hotspot

Not known to occur in any of the southern African biodiversity hotspots (Cape Floristic Region, Maputaland-Pondoland-Albany, or Succulent Karoo).

## Identification Keys

### Key to *Anasillomos* species

**Table:** 

1	Anepisternum dorso-medially white to light brown macrosetose (6–7 short macrosetae) (Fig. 3b); posterior half of anepisternum sparsely long white setose (Fig. 3b); T2–5 with apubescent stripe from antero-lateral corner to postero-median margin (stripes narrower on T3–5) (Fig. 3b); 2 light brown presutural and 3–4 light brown postsutural dorsocentral macrosetae (Fig. 3a); T2–4 with yellowish postero-lateral macrosetae (most pronounced on T2) among setae (Fig. 3b); wing hyaline throughout (Fig. 2a); antennal postpedicel 1.5x as long as scape and pedicel combined	*Anasillomos chrysopos*
–	Anepisternum dorso-medially only white setose (without short macrosetae) (Figs 5b, 6b); posterior half of anepisternum densely long white setose (Figs 5b, 6b); T2–5 with distinct lateral longitudinal dark grey pubescent stripe (Figs 5b, 6b, 7b); 1 white presutural and 3 white postsutural dorsocentral macrosetae (Figs 5a, 6a); T2–4 without postero-lateral macrosetae (regular setae present) (Figs 5b, 6b, 7b); wing hyaline but narrowly colored light brown along distal veins (Figs 5a, 6a); antennal postpedicel 2x as long as scape and pedicel combined	*Anasillomos juergeni* sp. n.

## Discussion

### Seasonal incidence

*Anasillomos* has been primarily collected between August (Southern Hemisphere late winter) and February (late summer) with the notable exception of January (see Table 1). The highest number of collecting events occurred in October–November (spring) with four and six collecting events, respectively. It should be noted that the entries for December represent a single collecting event at which both *Anasillomos* species were collected sympatrically. *A. chrysopos* appears to be flying earlier in the season (August–December) and *A. juergeni* sp. n. is flying later (October–February) (Table 1).

### Habitat, sympatry, and a central Namibian locality

On first examination, the two *Anasillomos* species appear to be occurring in different habitats in that *A. chrysopos* lives in savanna and Karoo biomes whereas *A. juergeni* sp. n. occurs in the Namib desert (Fig. 11). However, both species occur sympatrically in the Karoo of southern Namibia near Grünau. A single collecting event resulted in the caption of four females (two of each species) in December 1999, which were mentioned in Dikow and Londt (2000), but not assigned to any species. Only with the collection of several specimens of both sexes of *A. juergeni* sp. n. at Gobabeb and the comparison with type material, it became clear that there are two species flying sympatrically near Grünau.

Unfortunately, specimens previously studied by Londt (1983) and Dikow and Londt (2000) from the National Museum of Namibia in Windhoek (NMNW) were not available for study in this project. I am confident that the four specimens from around Windhoek deposited in NMNW represent *A. chrysopos* because a single female specimen from Hakos Mountains (south-west of Windhoek) in a savanna habitat has been studied (USNMENT00995574). The identification of the sole specimen from Awasib (25°23'00"S 015°39'00"E) in the central Namib desert as *A. chrysopos*, however, is questionable. Awasib lies a few kilometers east of the Namib sand dunes and approximately 100 km SSW of Sesriem from where *A. juergeni* sp. n. has been recorded. The proximity of Awasib to the large sand dunes and the collection in November possibly suggest that this specimen represents *A. juergeni* sp. n. and was therefore misidentified in Londt (1983) and Dikow and Londt (2000). This locality is highlighted in Fig. 11​ to provide a geographical reference and encourage future collecting along the eastern Namib desert transitioning into the savanna biome.

## Supplementary Material

Supplementary material 1 Natural-language species descriptions of Anasillomos as exported from Lucid Builder 3.5 in XML SDD format Data type: natural-language descriptions in SDD format
Brief description: Natural-language species descriptions of *Anasillomos* as exported from Lucid Builder 3.5 in XML SDD format.
File: oo_39101.sdd Dikow, T.

Supplementary material 2 Multi-entry, matrix-based identification key to Anasillomos as exported from Lucid Builder 3.5 in XML SDD format Data type: Multi-entry, matrix-based key in SDD format
Brief description: Multi-entry, matrix-based identification key to Anasillomos as exported from Lucid Builder 3.5 in XML SDD format
File: oo_39102.sdd T. Dikow

XML Treatment for Anasillomos

XML Treatment for Anasillomos chrysopos

XML Treatment for Anasillomos juergeni

## Figures and Tables

**Figure 2a.**
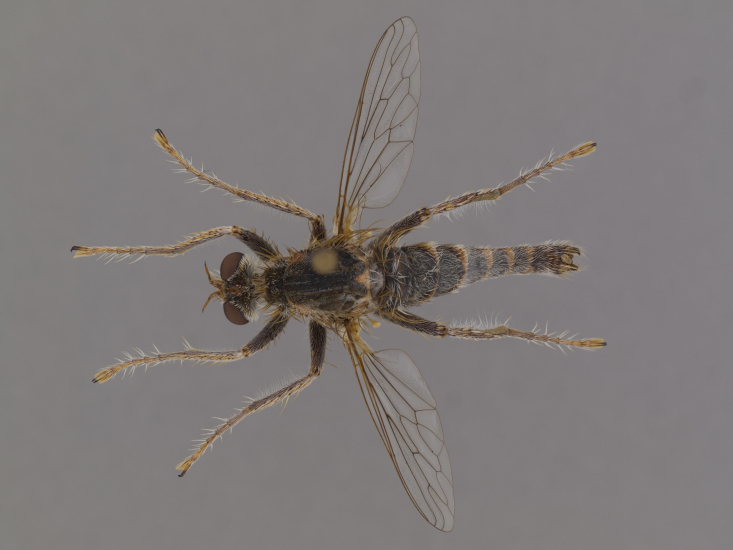
dorsal (Morphbank #850982)

**Figure 2b.**
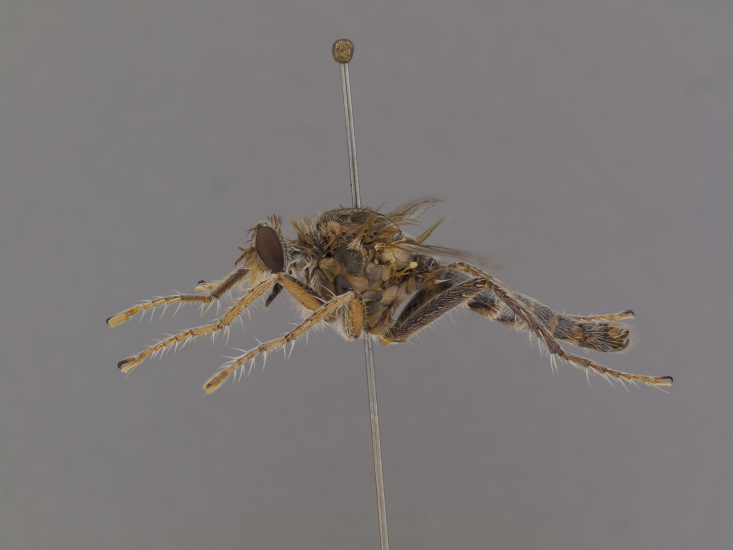
lateral (#850984)

**Figure 2c.**
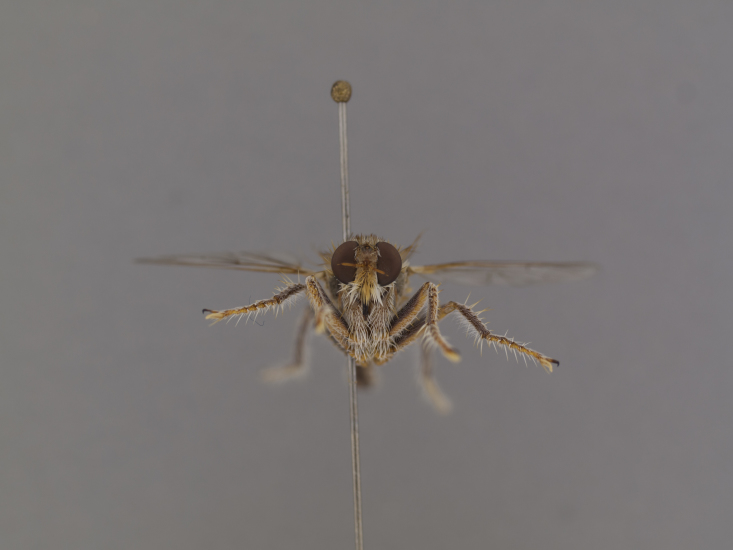
head anterior (#850986)

**Figure 3a.**
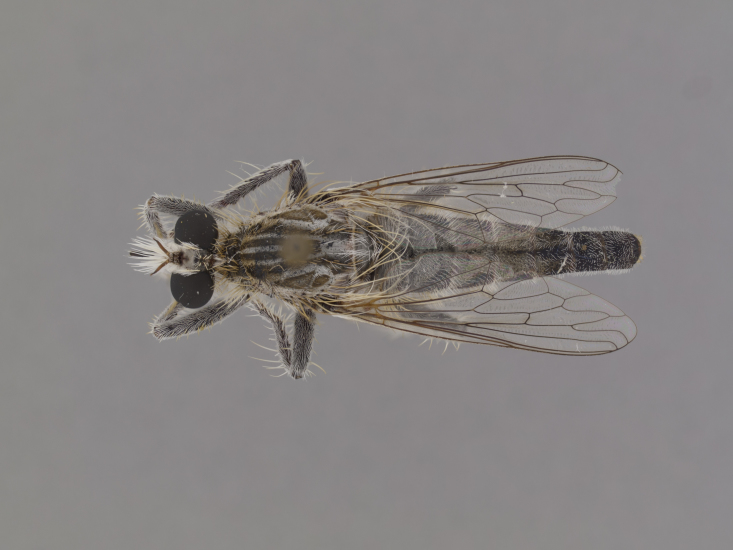
dorsal (Morphbank #850995)

**Figure 3b.**
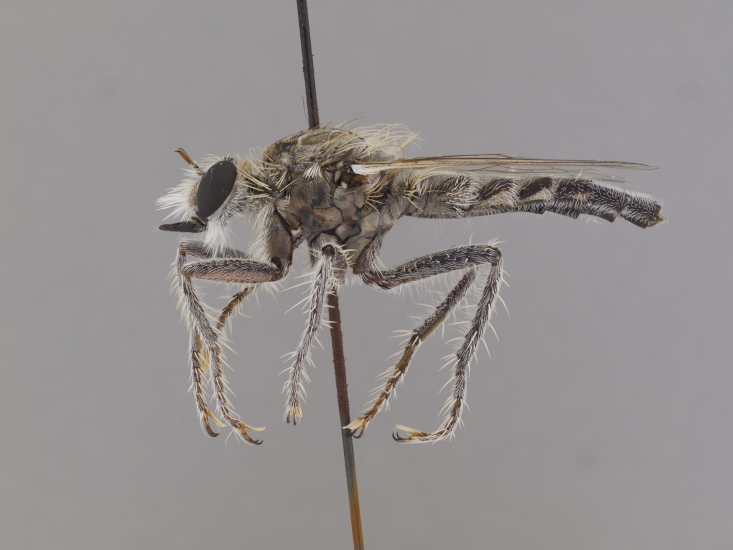
lateral (#850997)

**Figure 3c.**
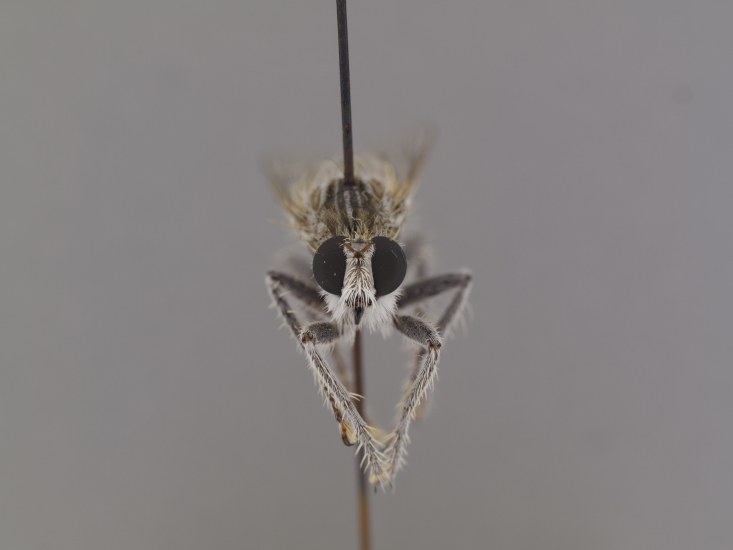
head anterior (#850999)

**Figure 4a.**
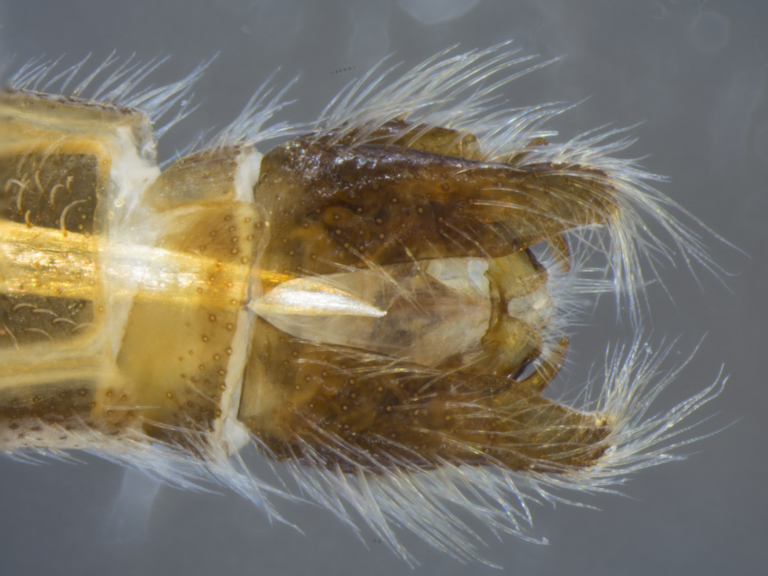
dorsal (Morphbank #850988)

**Figure 4b.**
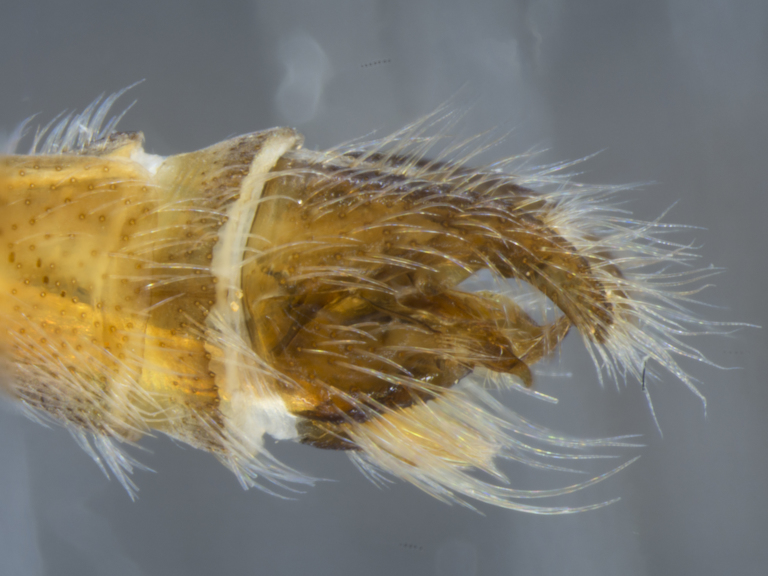
lateral (#850990)

**Figure 4c.**
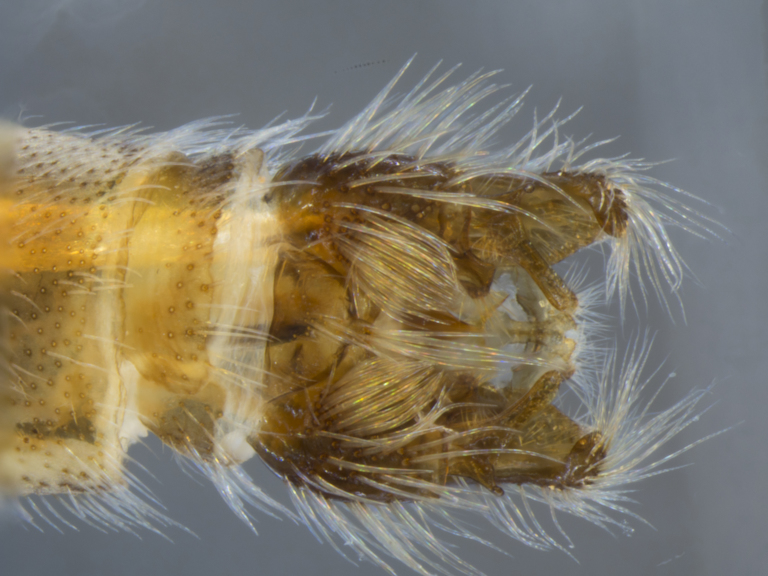
ventral (#850992)

**Figure 5a.**
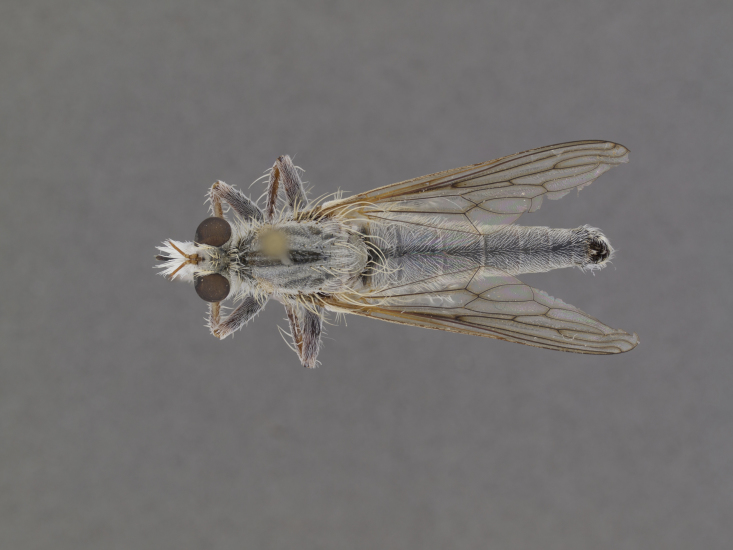
dorsal (Morphbank #850959)

**Figure 5b.**
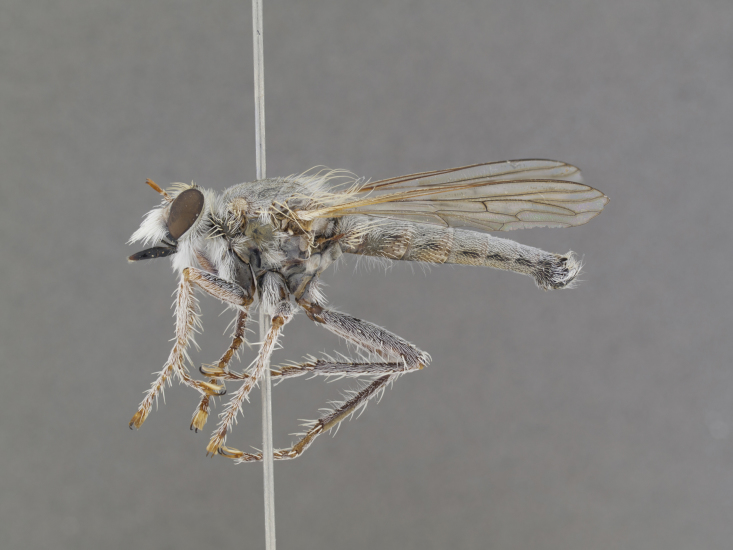
lateral (#850957)

**Figure 5c.**
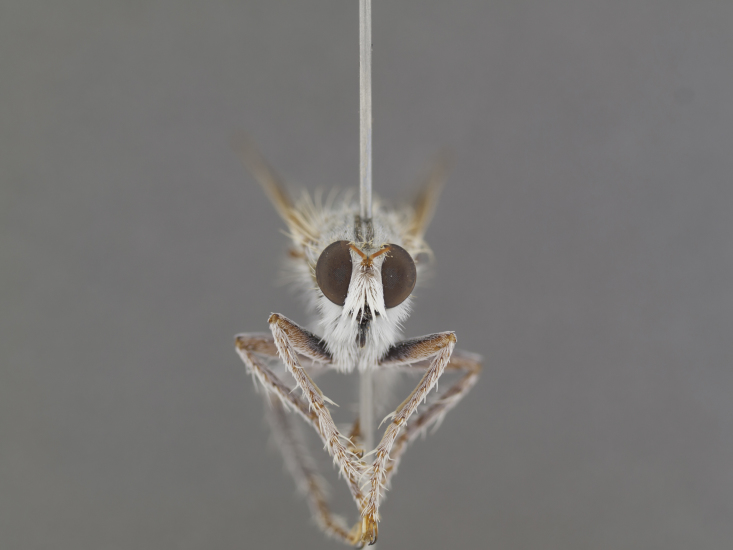
head anterior (#850963)

**Figure 5d.**
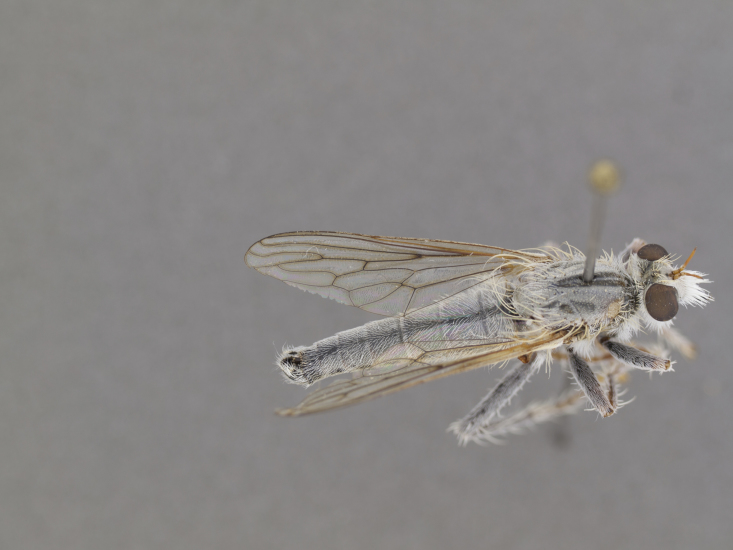
wing detail (#850961)

**Figure 6a.**
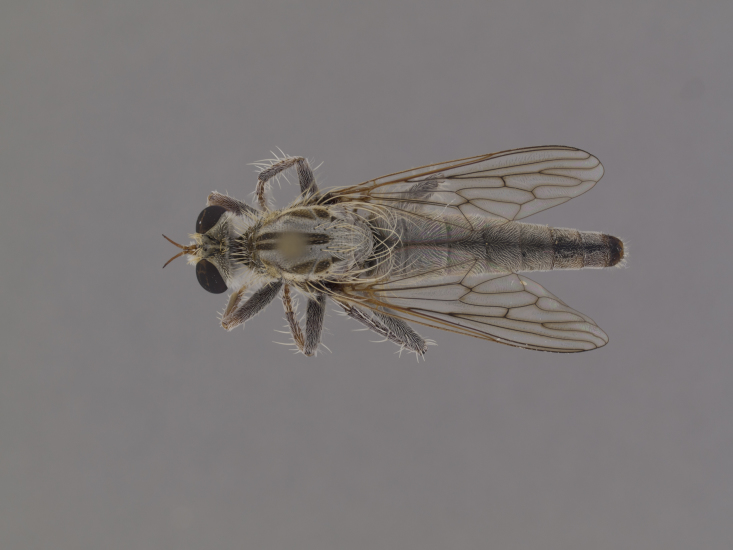
dorsal (Morphbank #850950)

**Figure 6b.**
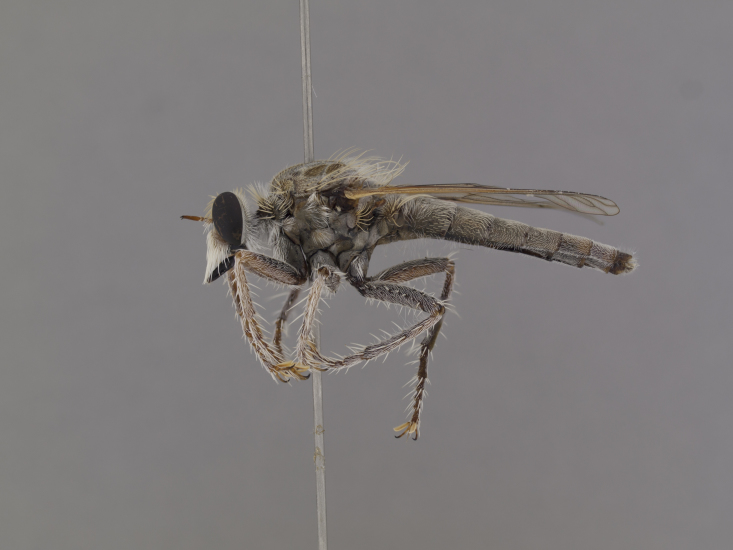
lateral (#850952)

**Figure 6c.**
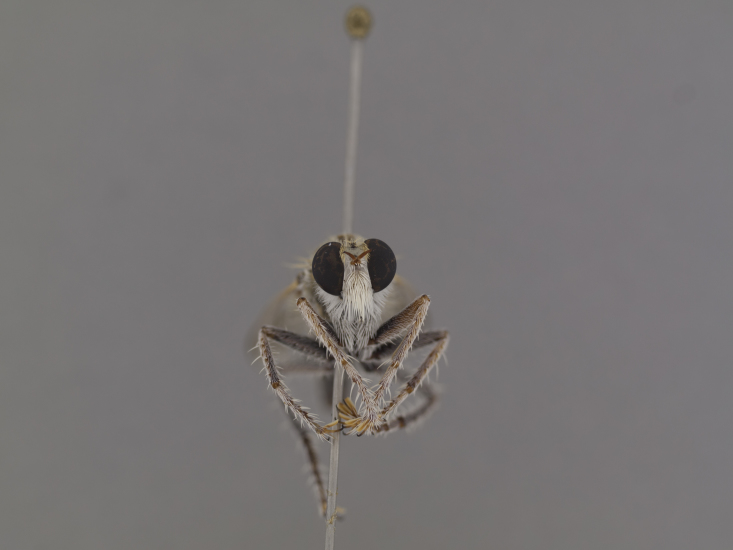
head anterior (#850954)

**Figure 7a.**
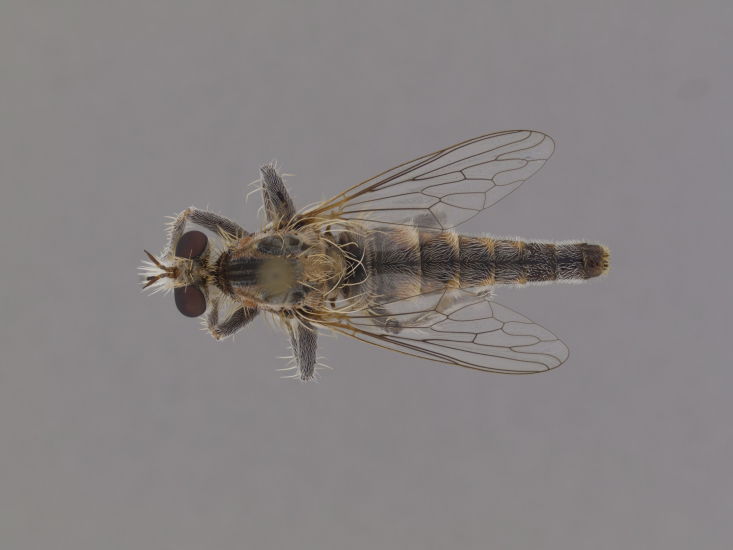
dorsal (Morphbank #850975)

**Figure 7b.**
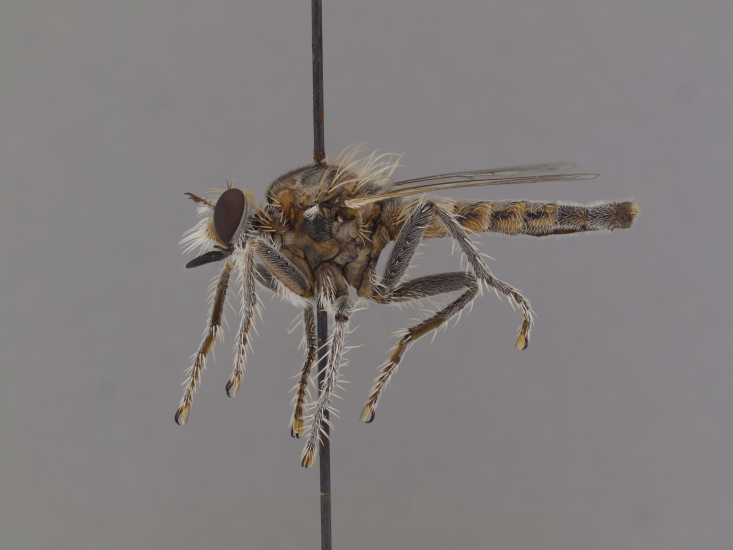
lateral (#850973)

**Figure 7c.**
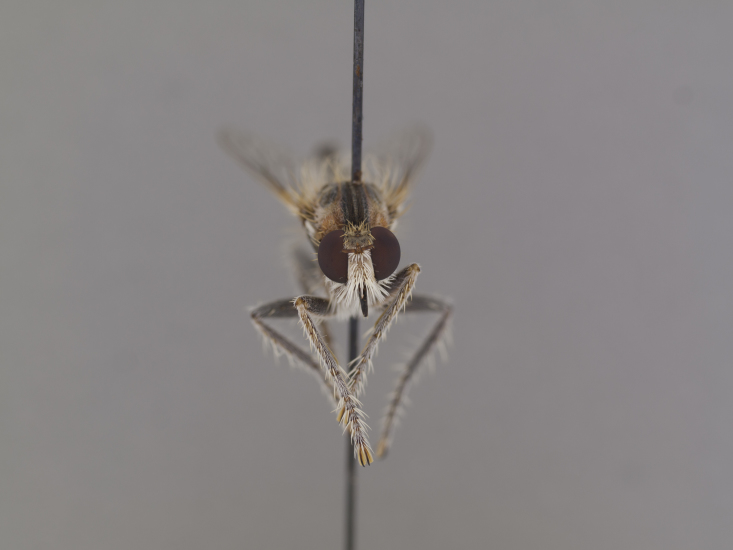
head anterior (#850977)

**Figure 8a.**
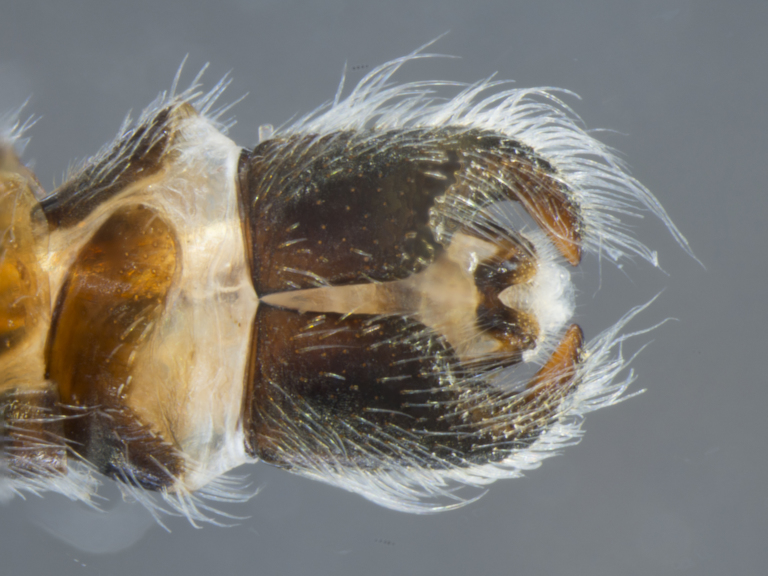
dorsal (Morphbank #850965)

**Figure 8b.**
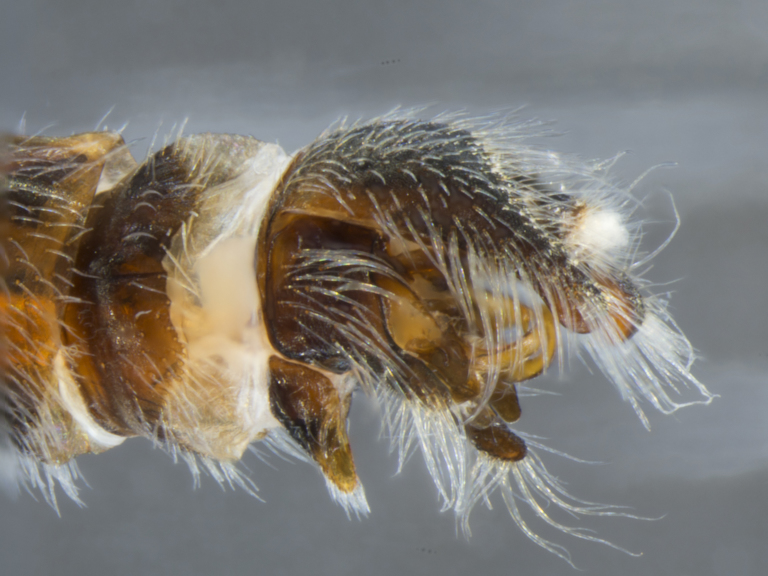
lateral (#850967)

**Figure 8c.**
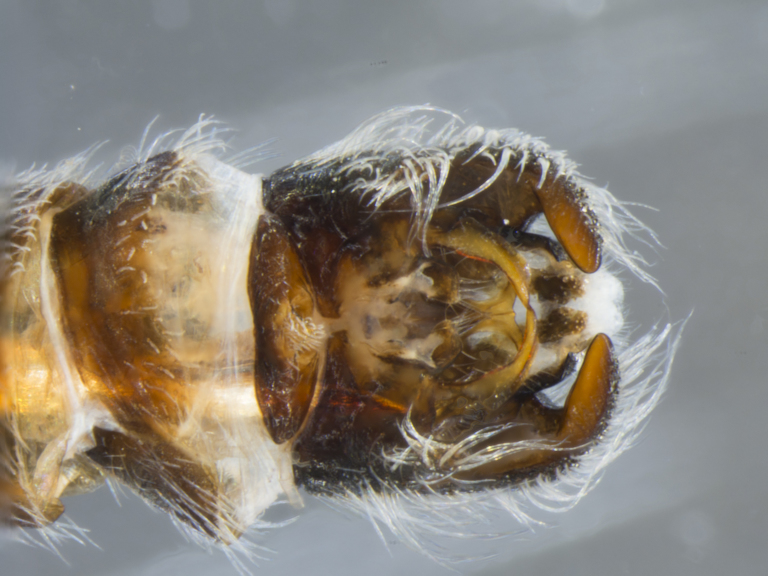
ventral (#850969)

**Figure 9a.**
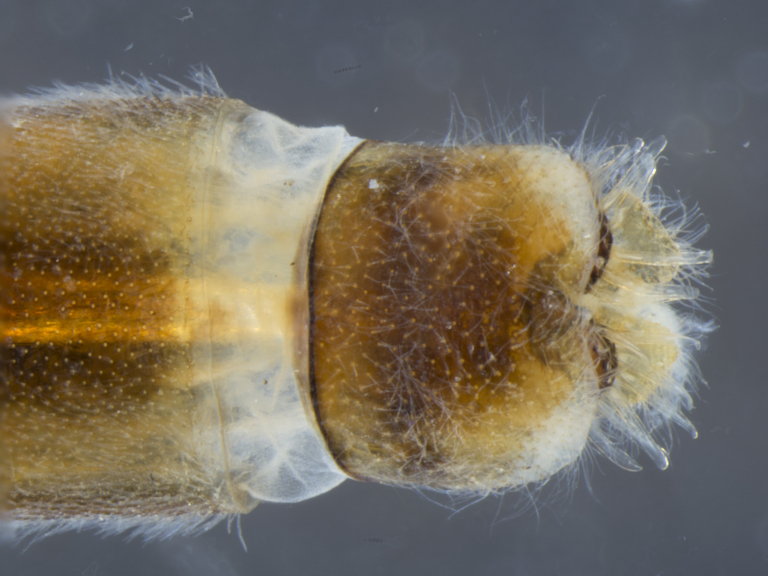
dorsal (Morphbank #851017)

**Figure 9b.**
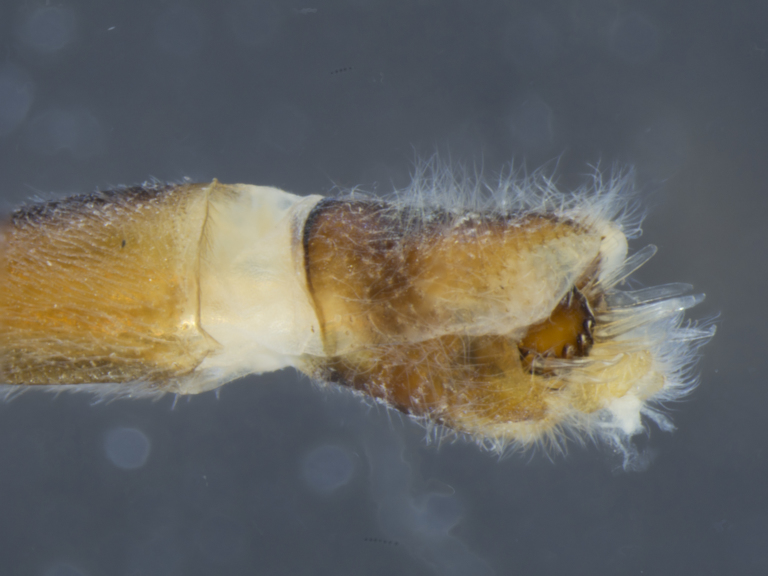
lateral (#851019)

**Figure 9c.**
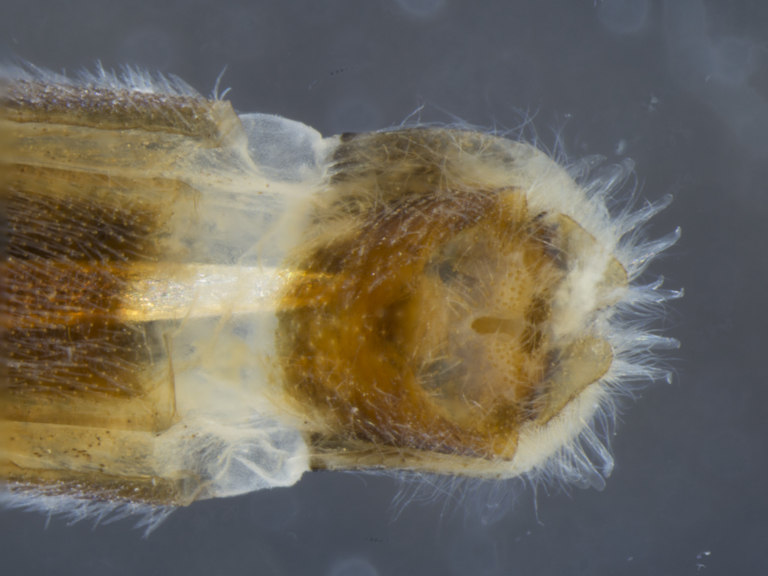
ventral (#851021)

**Figure 10a.**
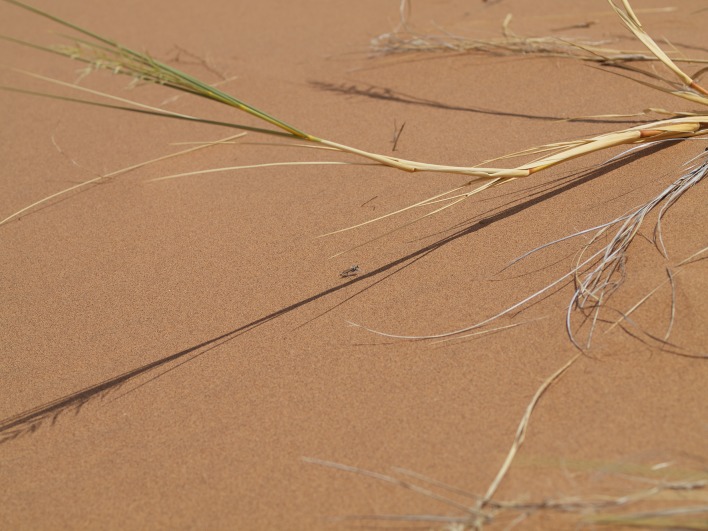
large, mostly bare sand dunes at Gobabeb (23°34'17"S 015°02'52"E) (note perching *A. juergeni* sp. n. assassin fly in center)

**Figure 10b.**
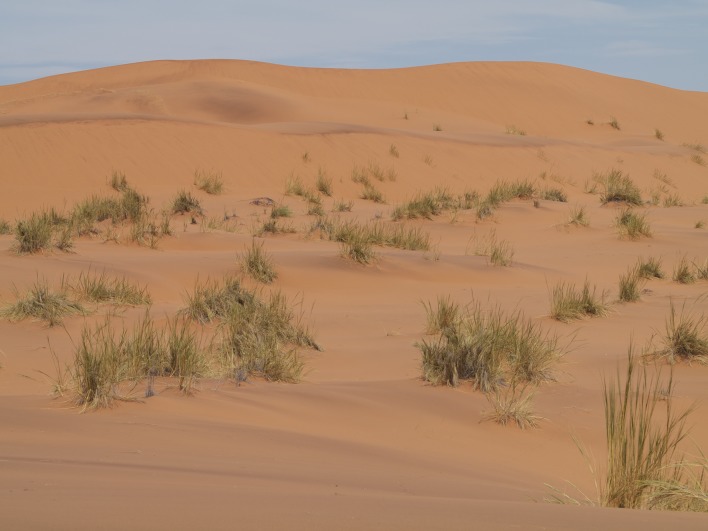
large, mostly bare sand dunes and partly vegetated interdune valleys at Gobabeb (23°34'17"S 015°02'52"E)

**Figure 10c.**
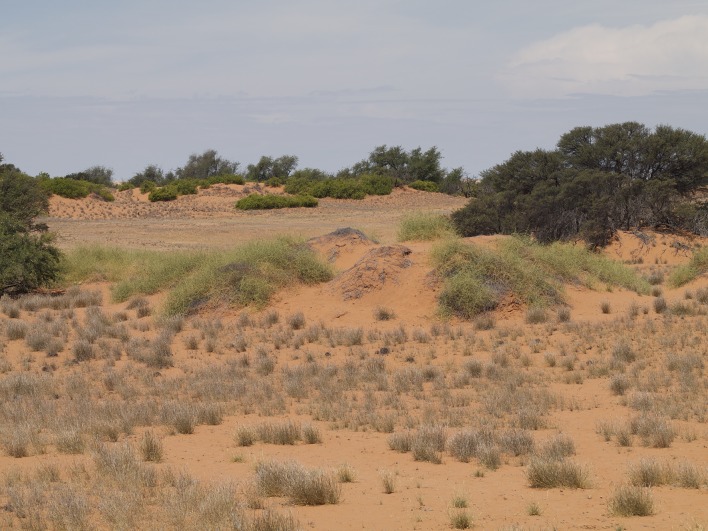
small, vegetated sand dunes at Gobabeb (23°33'50"S 015°02'01"E)

**Figure 10d.**
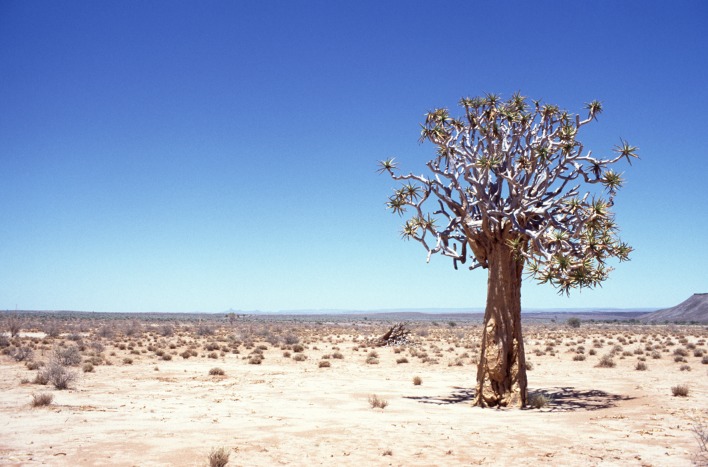
habitat near Grünau (27°30'45"S 017°50'01"E)

**Table 1.** Seasonal incidence of *Anasillomos* species. Numbers refer to specimens / collecting events in particular month. Note that the December records represent a single collecting event near Grünau where both species were collected sympatrically.

species	Jun	Jul	Aug	Sep	Oct	Nov	Dec	Jan	Feb	Mar	Apr	May
*A. chrysopos*			3/1	1	5/3	5/5	2/1					
*A. juergeni* sp. n.					1	1	2/1		11/4			
